# Small Molecule KRAS Agonist for Mutant KRAS Cancer Therapy

**DOI:** 10.1186/s12943-019-1012-4

**Published:** 2019-04-10

**Authors:** Ke Xu, Dongkyoo Park, Andrew T. Magis, Jun Zhang, Wei Zhou, Gabriel L. Sica, Suresh S. Ramalingam, Walter J. Curran, Xingming Deng

**Affiliations:** 1 Division of Cancer Biology, Department of Radiation Oncology, Emory University School of Medicine and Winship Cancer Institute of Emory University, Atlanta, GA 30322 USA; 2 Institute for Systems Biology, Seattle, WA 98109 USA; 3 Division of Hematology, Oncology and Blood & Marrow Transplantation, Department of Internal Medicine, Holden Comprehensive Cancer Center, University of Iowa Carver College of Medicine, Iowa City, IA 52242 USA; 4 Department of Hematology and Medical Oncology, Emory University School of Medicine and Winship Cancer Institute of Emory University, Atlanta, GA 30322 USA; 5 Department of Pathology and Laboratory Medicine, Emory University School of Medicine and Winship Cancer Institute of Emory University, Atlanta, GA 30322 USA

**Keywords:** KRAS, Agonist, Apoptosis, Autophagy, NSCLC, Therapy

## Abstract

**Background:**

Lung cancer patients with KRAS mutation(s) have a poor prognosis due in part to the development of resistance to currently available therapeutic interventions. Development of a new class of anticancer agents that directly targets KRAS may provide a more attractive option for the treatment of KRAS-mutant lung cancer.

**Results:**

Here we identified a small molecule KRAS agonist, KRA-533, that binds the GTP/GDP-binding pocket of KRAS. In vitro GDP/GTP exchange assay reveals that KRA-533 activates KRAS by preventing the cleavage of GTP into GDP, leading to the accumulation of GTP-KRAS, an active form of KRAS. Treatment of human lung cancer cells with KRA-533 resulted in increased KRAS activity and suppression of cell growth. Lung cancer cell lines with KRAS mutation were relatively more sensitive to KRA-533 than cell lines without KRAS mutation. Mutating one of the hydrogen-bonds among the KRA-533 binding amino acids in KRAS (mutant K117A) resulted in failure of KRAS to bind KRA-533. KRA-533 had no effect on the activity of K117A mutant KRAS, suggesting that KRA-533 binding to K117 is required for KRA-533 to enhance KRAS activity. Intriguingly, KRA-533-mediated KRAS activation not only promoted apoptosis but also autophagic cell death. In mutant KRAS lung cancer xenografts and genetically engineered mutant KRAS-driven lung cancer models, KRA-533 suppressed malignant growth without significant toxicity to normal tissues.

**Conclusions:**

The development of this KRAS agonist as a new class of anticancer drug offers a potentially effective strategy for the treatment of lung cancer with KRAS mutation and/or mutant KRAS-driven lung cancer.

**Electronic supplementary material:**

The online version of this article (10.1186/s12943-019-1012-4) contains supplementary material, which is available to authorized users.

## Background

RAS family genes, including HRAS, KRAS and NRAS, are the most common oncogenes in human cancer, and encode extremely similar proteins made up of chains of 188 to 189 amino acids. The sequences and structural features of these three proteins are highly conserved, except for their carboxyl-terminal domains and post-translational lipid modifications [1, 2]. HRAS, KRAS and NRAS are regulated in a similar manner within the cell. The RAS genes encode monomeric GTPases that function as molecular switches in signal transduction pathways regulating cell proliferation, differentiation and survival in mammalian cells [1]. Mutations that can constitutively activate RAS have been found in 20% ~ 25% of all human cancers [3]. KRAS has the highest mutation rate compared to HRAS and NRAS in various types of cancers [4, 5]. KRAS is a proto-oncogene and its gene product was first found as a p21 GTPase. KRAS binds to GTP in its active state and possesses an intrinsic enzymatic activity which cleaves the terminal phosphate of the nucleotide, converting it to GDP. Upon conversion of GTP to GDP, KRAS is deactivated. The rate of conversion is usually slow, but can be increased dramatically by an accessory GTPase-activating protein (GAP) [4]. In turn, KRAS can bind to guanine nucleotide exchange factors (GEFs) (such as SOS), which force the release of bound nucleotide (GDP). GTP binding enables several residues, primarily in the switch I region (residues 30–40) and switch II region (residues 60–70), to adopt a conformation that permits KRAS effector proteins to bind; these switches are regulated by GAPs and GEFs [6, 7]. In mammalian cells, endogenous KRAS proteins are predominantly in the GDP state and activation is transient [8]. However, the common oncogenic mutations in KRAS proteins interfere with GTP hydrolysis, resulting in proteins that remain in the active GTP state and continue to transmit signals to effector pathways [1]. Thus, KRAS acts as a molecular on/off switch. Once it is turned on, it recruits and activates proteins necessary for the propagation of signaling of growth factors and other receptors, such as c-Raf and PI3K [9–12].

Somatic KRAS mutations are found at high rates in leukemia [13, 14], colorectal cancer [15], pancreatic cancer [16] and non-small cell lung cancer (NSCLC) [17]. In NSCLC, KRAS mutation is observed in up to 30–40% of cases [11, 12, 18]. The most common KRAS mutations include G12C, G12D, G12R, G12S, G12 V, G13D and Q61H [19, 20]. Beyond the most common hotspot alleles in exons 2 and 3, mutations in exon 4 of KRAS, including K117 N and A146T, have also been found in patients with colorectal cancer [21, 22]. KRAS mutations constitutively activate KRAS and subsequently its downstream Raf/MEK/ERK1/2 and PI3K/PIP3/AKT survival pathways in various cancers, including lung cancer [9–12]. However, over the past two decades, evidence has gradually accumulated to support a paradoxical role for RAS proteins in the initiation of cell death pathways [1, 23–25]. Hyperactive RAS forces cells into the pathway of programmed cell death [26]. Vitamin C treatment selectively kills mutant KRAS expressing tumor cells, but not wild-type KRAS containing cells [27]. Interestingly, either glucose withdrawal or glucose-mediated hyperactivation of RAS is able to trigger apoptosis [26, 28]. RAS oncogenes trigger apoptosis only under specific conditions [26]. Thus, manipulation of the opposing functions of KRAS in cell proliferation/survival versus cell death should be an attractive approach to develop new strategies for the treatment of various types of cancers, especially those with mutant KRAS. Currently, there are no effective targeted therapies for patients with KRAS mutant cancers [29, 30]. KRAS has been considered an “undruggable” target and it is difficult to inhibit its intracellular activity [5, 31] for the following reasons [31]: first, whether KRAS adopts an active or inactive form depends on its GTP or GDP binding status rather than it being a substrate of catalytic reactions; second, there is a picomolar affinity between KRAS and GTP while micromolar concentrations of GTP exist in cancer cells; third, KRAS lacks a sufficiently large and deep hydrophobic pocket for small molecule binding, aside from the challenging nucleotide-binding site [4–6, 32]. Therefore, numerous efforts made by industry and academic laboratories have failed to design a drug to inhibit KRAS activity in cancer cells by directly targeting KRAS. Since intracellular KRAS activity is difficult to disrupt, and activated KRAS has been demonstrated to trigger death pathways [1], changing the nature of KRAS signaling from pro-survival to pro-death by directly targeting KRAS in cancer cells may represent an entirely new strategy for cancer therapy. Here we identified KRA-533 as a novel KRAS agonist that binds to the GTP/GDP binding pocket in the KRAS protein to prevent GTP cleavage, resulting in the accumulation of constitutively active GTP-bound KRAS that triggers both apoptotic and autophagic cell death pathways in cancer cells, leading to potent suppression of mutant KRAS lung cancer in vitro and in animal models.

## Methods

### Materials

Small molecule NSC112533 (KRA-533) was obtained from the Drug Synthesis and Chemistry Branch, Developmental Therapeutic Program, Division of Cancer Treatment and Diagnosis, National Cancer Institute (NCI, Bethesda, MD; http://dtp.nci.nih.gov/RequestCompounds). KRAS was purchased from Thermo Fisher Scientific (Rockford, IL). Anti-GFP, PARP and Beclin-1 antibodies, and KRAS shRNA (sc-35,731-SH) were purchased from Santa Cruz Biotechnology (Santa Cruz, CA). ERK, p-ERK (Thr202/Tyr204), LC3I/II and active/cleaved caspase-3 antibodies were purchased from Cell Signaling Technology (Danvers, MA). Protein Thermal Shift™ Dye Kit and Raf-1-RBD beads for pull-down of active RAS and Detection Kit were purchased from Thermo Fisher Scientific (Rockford, IL). Purified recombinant RASA1 and RASGRP1 proteins were purchased from OriGene Technologies (Rockville, MD). [γ-^35^S] GTP was purchased from PerkinElmer (Waltham, MA). Oligonucleotides were purchased from Integrated DNA Technologies (Coralville, IA). Chloroquines, 3-methyladenine (3-MA) and JC-1 were purchased from MedChemExpress (Monmouth Junction, NJ). All other reagents used were obtained from commercial sources unless otherwise stated.

### Cell lines and cell culture

Normal lung epithelial and lung cancer cell lines were obtained from the American Type Culture Collection. NSCLC cell line A549 was cultured in Dulbeccos’ Modified Eagles’ Medium (DMEM)/F-12medium supplemented with 10% FBS as described [33]. HCC827, H292, H1975, H322, Calu-1, H157, H358 and H1792 were cultured in RPMI-1640 medium supplemented with 5% FBS and 5% BS. These cell lines were used for the described experiments without further authentication.

### Protein analysis by Western blot

Cells were lysed with EBC buffer at 4 °C for 30 min. After sonication, samples were centrifuged at 12000×g for 15 min, and supernatant was quantified using a BCA protein assay kit (Thermo Fisher Scientific, Rockford, IL). Equal amounts of protein samples were separated by SDS-PAGE, and transferred to nitrocellulose membranes using an electro-blotting apparatus (Bio-Rad, Hercules, CA, USA). The membranes were blocked in blocking buffer (PBS-T plus 5% skimmed milk), and incubated with primary antibodies overnight at 4 °C. The membranes were washed with PBS-T and incubated with horseradish peroxidase-conjugated secondary antibodies for 0.5 h at room temperature. Protein expression was visualized using the ECL kit (Amersham Biosciences) as described [34].

### Plasmids and transfections

GFP-LC3 plasmid was kindly provided by Dr. William A. Dunn (University of Florida). Human KRAS-WT, G12C, G12D and G13D cDNA (s) in pEGFP-C3 expression plasmids were provided by Dr. Wei Zhou (Emory University, USA). For generation of single KRAS point mutations at codons 17 (S to A) and 117 (K to A), primers were synthesized as follows: S17A, Forward: 5′-CTG GTG GCG TAG GCA AGG CT GCC TTG ACG ATA CAG-3′, Reverse: 5′-CTG TAT CGT CAA GGC AGC CTT GCC TAC GCC ACC AG-3′; K117A, Forward: 5′-CCT ATG GTC CTA GTA GGA AAT GCA TGT GAT TTG CCT TCC AGA AC-3′, Reverse: 5′-GTT CTG GAA GGC AAA TCA CAT GCA TTT CCT ACT AGG ACC ATA GG-3′. These KRAS single point mutations were created using the QuikChange™ Site-Directed Mutagenesis Kit (Agilent Technologies, CA) according to the manufacturer’s instructions [35]. Each single mutant was confirmed by sequencing of the cDNA. KRAS mutants were transfected into cells using Lipofectamine 2000 (Invitrogen) according to the manufacturer’s instructions.

### Measurement of intracellular KRAS activity

The cellular activity of the KRAS protein was measured using active RAS pull-down and detection kit according to the manufacturer’s instructions (Thermo Fisher Scientific, Rockford, IL) as described [36, 37]. GTP-bound forms of KRAS were pulled-down from cell lysates using GST-Raf-1 RBD beads. After washing, loading buffer was added to samples and boiled for 5 min, followed by SDS-PAGE and Western blot using KRAS antibody.

### Production of purified KRAS protein

WT, G12C, G12D, G13D, S17A or K117A KRAS mutant cDNA were cut from pEGFP-C3 constructs with BamH I and Hind III, and cloned into pET-20b (+) vector (His-tag) (GE Healthcare) between BamH I and Hind III. The recombinant KRAS proteins were expressed in *Escherichia coli* (BL21 (DE3)) and purified as described [38]. Briefly, after bacterial growth to an absorbance (OD) at 600 nm of 0.4–0.6 in Terrific Broth containing 30 mg/L kanamycin at 37 °C, induction was carried out at 18 °C using 0.5 mM isopropyl-b-D-thiogalactoside (IPTG), and growth was continued at 18 °C for about 18 h. The bacteria were collected by centrifugation, and the obtained pellet either stored at − 80 °C or used freshly for the subsequent steps. His-tagged-KRAS was purified using 5-ml Hi Trap Ni2 + −Sepharose column equilibrated with buffer A containing 20 mM imidazole. Bound proteins were eluted with a linear concentration gradient of imidazole (i.e. 50 and 350 mM) in 50 ml buffer A. Fractions containing KRAS protein were pooled, dialyzed against buffer B (20mMTris-HCl, pH 8.0, 100 mM NaCl, 10% (*v*/v) glycerol and 1 mM DTT) and loaded onto a 1-ml Hi Trap Q Sepharose FF column. Bound proteins were eluted with a linear concentration gradient of NaCl (i.e. 50 and 500 mM) in 12 ml buffer B. Purified proteins were stored at − 80 °C. The concentration of purified proteins was determined by Bradford assay. The values obtained by Bradford assay were divided by the predicted molecular mass of the appropriate protein to calculate its molar concentration.

### GDP-GTP exchange assay in cell-free system

In vitro GDP-GTP exchange assay was performed as described [39]. Briefly, purified WT or mutant KRAS proteins (600 nM) were incubated with purified GEF (RASGRP1, 180 nM each) and GAP (RASA1, 180 nM each) proteins in buffer B [50 mM Tris·HCl (pH 7.4), 50 mM NaCl, 5 mM MgCl2, 1 mM DTT, and 20 mM imidazole] containing [γ-^35^S]-GTP (11 μM) in the absence or presence of increasing concentrations of KRA-533 at 25 °C for 60 min. After intensively washing, KRAS activity was quantified by liquid scintillation counting.

### Measurement of KRA-533/KRAS binding by thermal shift assay

Thermal shift assay was performed using the Protein Thermal Shift Dye Kit (Thermo Fisher Scientific, Rockford, IL) as described [40]. Purified WT and KRAS mutant proteins were incubated with increasing concentrations of KRA-533 in Protein Thermal Shift buffer containing Thermal Shift Dye at room temperature, followed by measurement of the fluorescence release using Real-Time PCR Systems (Applied Biosystems). Data were analyzed using Protein Thermal Shift Software v1.0 (Life Technologies) [40].

### Clonogenic survival assay

Cells were seeded in 6-well plates or cell culture dishes. After 12 h, cells were treated with KRA-533 (10 μM). The medium was replaced with fresh medium containing KRA-533 every 3 days. After 10 days of treatment, the medium was removed and cell colonies were stained with crystal violet (0.1% in 20% methanol). Surviving colonies were counted and the surviving fraction (SF) was calculated using the formula SF = treatment colony numbers/control colony numbers after at least three independent experiments as described [41].

### Silencing of KRAS

Human KRAS shRNA and Ctrl shRNA in pRS shRNA vectors were obtained from Santa Cruz Biotechnology (Santa Cruz, CA). Human KRAS shRNA Plasmid is a pool of 3 target-specific lentiviral vector plasmids each encoding 19–25 nt (plus hairpin) shRNAs designed to specifically knock down KRAS gene expression. Sc-35,731-SHA: hairpin sequence, 5′ GAT CCG GAA GCA AGT AGT AAT TGA TTC AAG AGA TCA ATT ACT ACT TGC TTC CTT TTT 3′; sc-35,731-SHB: hairpin sequence: 5′ GAT CCC TAG AAC AGT AGA CAC AAA TTC AAG AGA TTT GTG TCT ACT GTT CTA GTT TTT 3′, sc-35,731-SHC: hairpin sequence, 5′ GAT CCG AAC CTT TGA GCT TTC ATA TTC AAG AGA TAT GAA AGC TCA AAG GTT CTT TTT 3′. For pseudovirus production, KRAS shRNA plasmids were cotransfected into 293FT cells with a retrovirus packaging plasmid mixture (Agilent technologies, CA) using the Nanojuice transfection kit (EMD Chemical, Inc.). After 48 h, the virus-containing media were harvested by centrifugation at 20,000×g. Human lung cancer A549 cells were infected with virus-containing media in the presence of polybrene (8 μg/ml) for 24 h. Stable positive clones were selected using 1 μg/ml puromycin. The silencing efficiency of the targeted KRAS gene was confirmed by Western blotting.

### Apoptosis assay

Apoptotic and viable cells were detected using an Annexin V/PI kit (BD Pharmingen, CA) according to the manufacturer’s instructions. The percentage of viable cells or apoptotic cells was determined by fluorescence-activated cell sorter (FACS) analysis as described [42, 43].

### Cell proliferation assay

NSCLC cells were seeded in 96-well plates at 6 × 10^3^ cells per well and allowed to grow overnight. Then, cells were treated with KRA533, followed by analysis of cell proliferation using MTS Cell Proliferation Colorimetric Assay Kit from Promega (Madison, WI) according to the manufacturer’s instructions. The optical density (OD) values were analyzed by measuring the absorbance at 490–500 nm with microplate reader (Perkin-Elmer, Waltham, MA, USA).

### Measurement of autophagy with GFP-LC3 construct

GFP-LC3 constructs were transfected into human lung cancer cells, followed by treatment with increasing concentrations of KRA-533 for 48 h. Autophagic cells were visualized by Axioplan Zeiss microscope (Zeiss, German) and quantified as described [44].

### Caspase 3 activity assay

Cells were treated with KRA-533, followed by analysis of caspase 3 activity using a Caspase 3 Colorimetric Assay Kit from Abcam (Cambridge, MA) according to the manufacturer’s instructions. OD values were analyzed by measuring the absorbance at 400–405 nm with microplate reader (Perkin-Elmer, Waltham, MA, USA).

### Lung cancer xenografts and treatments

Six-week-old female Nu/Nu nude mice were purchased from Harlan and housed under pathogen-free conditions in micro isolator cages. All animal treatments were undertaken in accordance with protocols approved by the Institutional Animal Care and Use Committee (IACUC) at Emory University (Atlanta, GA). 3 × 10^6^ A549 cells in Hanks’ Balanced Salt Solution (HBSS, Gibco) were injected into subcutaneous tissue at the flank region of nude mice. The tumors were allowed to grow to an average volume of about 250 mm^3^ before initiation of therapy as described [45]. Mice were treated with KRA-533 intraperitoneally (i.p.). During treatment, tumor volume (*V*) was measured by caliper measurements once every 2 days and calculated with the formula: *V* ¼ (L× W^2^)/2 (L is the length and W is the width). Mice were sacrificed by inhaled CO_2_ at the end of treatment. Harvested tumors were weighed and immediately fixed in formalin for immunohistochemistry.

### Immunohistochemistry (IHC) analysis

Tumors were harvested, fixed in formalin and embedded in paraffin. Representative sections from paraffin-embedded tumor tissues were analyzed by IHC staining using anti-active caspase 3 (1:100), LC3-II (1:100) or pERK (1100) antibodies. Active caspase 3-positive cells, LC3-II-positive cells or pERK-positive cells in tumor tissues were scored at 400× magnification. The average number of positive cells per 0.0625 mm^2^ area was determined from three separate fields in each of three independent tumor samples as described [45, 46].

### Genetically engineered lox-stop-lox (LSL*)*-KRAS^G12D^ and LSL-KRAS^G12D^ LKB1^fl/fl^ (KL) mouse models, treatment and tumor burden quantification

Lox-stop-lox (LSL*)*-KRAS^G12D^ and LSL-KRAS^G12D^ LKB1^fl/fl^ (KL) were generated as previously described [47, 48]. All studies were performed on protocols approved by the Emory University IACUC. After cre-adenovirus infection, 10 weeks for KRAS^G12D^ mice or 6 weeks for KL mice, mice were treated with KRA-533 intraperitoneally (i.p) for 3 months (KRAS^G12D^ mice) or 8 weeks (KL mice). Mice were euthanized with CO_2_ asphyxiation. After lung perfusion with PBS, the left lung lobes were harvested from mice in control and KRA-533 treated groups and immediately fixed in 10% neutral buffered formalin (Fisher Scientific, Kalamazoo, MI), horizontally cut into three equal parts and embedded in paraffin blocks. Three parts of lung tissues representing different regions of the lung were vertically put into paraffin blocks for hematoxylin-eosin (H&E) staining. Lung tissue samples were sectioned at 3 μm three times for placement of slides and stained with H&E. H&E stained samples were scanned using Nano Zoomer 2.0-HT (Hamamatsu, Japan) and images were analyzed using ImageScope viewing software (Leica Biosystems, Buffalo Grove, IL). Tumor numbers were counted under a microscope and tumor area was quantified using Openlab modular imaging software (PerkinElmer, Waltham, MA) as previously described [46, 48].

### Mouse blood analysis

Whole blood (250 mL) was collected in EDTA-coated tubes via cardiac puncture of anesthetized mice for hematology studies. Specimens were analyzed for white blood cells (WBC), red blood cells (RBC), platelets (PLT), alanine aminotransferase (ALT), aspartate aminotransferase (AST), and blood urea nitrogen (BUN) in the Clinical Pathology Laboratory at the University of Georgia (Athens, GA).

### Statistical analysis

All data are presented as mean ± standard deviation (SD) from at least three independent experiments. The statistical significance of differences between groups was analyzed with 2-tailed *t* test. We chose the sample size to detect a minimum effect size of 1.5 with at least 80% power and a type I error of 0.05 for each comparison. A value of *P <* 0.05 was considered statistically significant.

## Results

### Screening of small molecules that target the GTP/GDP binding pocket of KRAS

**Fig. 1:**
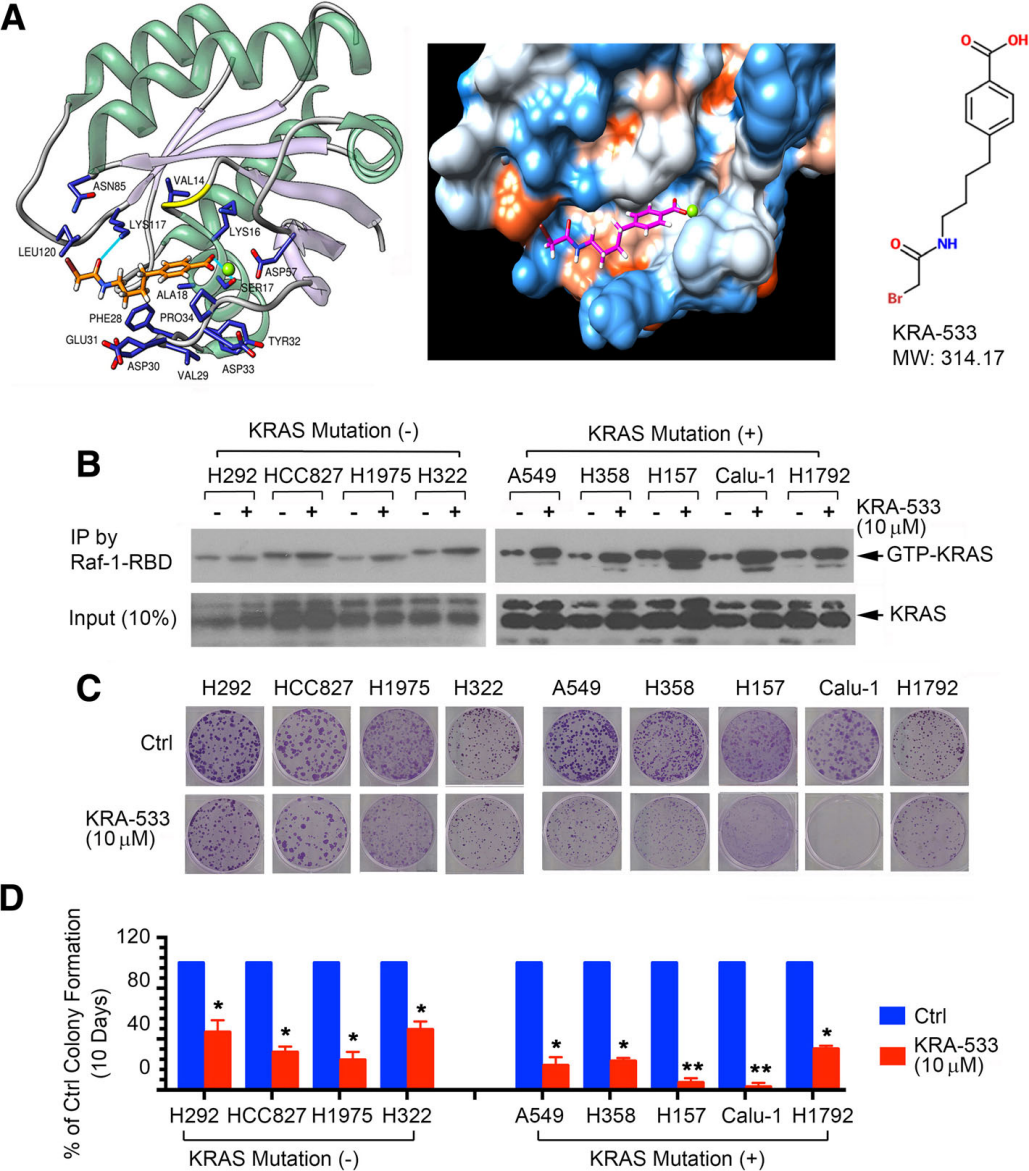
KRA-533 activates KRAS in association with growth inhibition of NSCLC cells. (**a**) Structural modeling of KRA-533 in the GTP/GDP-binding pocket of KRAS protein. (**b**) Various NSCLC cells with or without KRAS mutation were treated with KRA-533 (10 μM) for 48 h. KRAS-GTP (active form of KRAS) was pulled down by Raf-1-RBD, followed by Western blot using KRAS antibody. (**c**) and (**d**) Various NSCLC cells were treated with KRA-533 (10 μM) for 10 days, followed by colony formation assay with quantification. Error bars represent ± SD. *P < 0.05, **P < 0.01, by 2-tailed *t* test

A library containing ~ 300,000 small molecules from the National Cancer Institute (NCI) was employed to dock the GTP/GDP binding pocket of KRAS (PDB ID code: 4EPT) using the University of California, San Francisco (UCSF) DOCK 6.1 program suite as we previously described [49–51]. The small molecules were ranked according to their energy scores. Top 500 small molecules with predicted binding energies were selected for screening of cytotoxicity in human lung cancer cells by sulforhodamine B (SRB) assay as previously described. Among these small molecules, the compound NSC112533 (C_13_H_16_BrNO_3_, molecular weight [MW] 314.17) had the most potent activity against human lung cancer cells. We named this lead compound small-molecule KRAS agonist-533 (KRA-533). The molecular modeling of KRA-533 in complex with KRAS GTP/GDP binding pocket is shown in Fig. 1A.

To test whether KRA-533 regulates KRAS activity in lung cancer cells, human lung cancer cell lines with or without KRAS mutation were treated with KRA-533 (10 μM) for 48 h. GTP-KRAS (active form of KRAS) was pulled down using Raf-1-RBD beads, followed by Western blot using KRAS antibody. KRA-533 enhanced KRAS activity in most human lung cancer cell lines tested, except H292. Intriguingly, KRA-533 enhanced KRAS activity to a greater extent in cell lines bearing KRAS mutation than in cell lines without KRAS mutation (Fig. 1B). Colony formation analysis following 10-day’s treatment revealed that human lung cancer cell lines with KRAS mutation (i.e. A549, H358, H157, Calu-1 and H1972) were relatively more sensitive to KRA-533-mediated cell growth suppression than those without KRAS mutation (i.e. H292, HCC827, H1975 and H322) (Fig. 1C and D). Cell proliferation was also measured following treatment of cells for 48 h using MTS Cell Proliferation Colorimetric Assay Kit. Treatment of various NSCLC cells with KRA-533 resulted in suppression of cell proliferation. Similarly, A549, H157 and Calu-1 cell lines bearing KRAS mutation were more sensitive to KRA-533 than H292 cells without KRAS mutation (Additional file 1**:** Figure S1). These findings suggest that KRA-533 may be more suitable to treat mutant KRAS lung cancer.

### KRA-533 directly binds and activates WT and mutant KRAS in an in vitro cell-free system

**Fig. 2:**
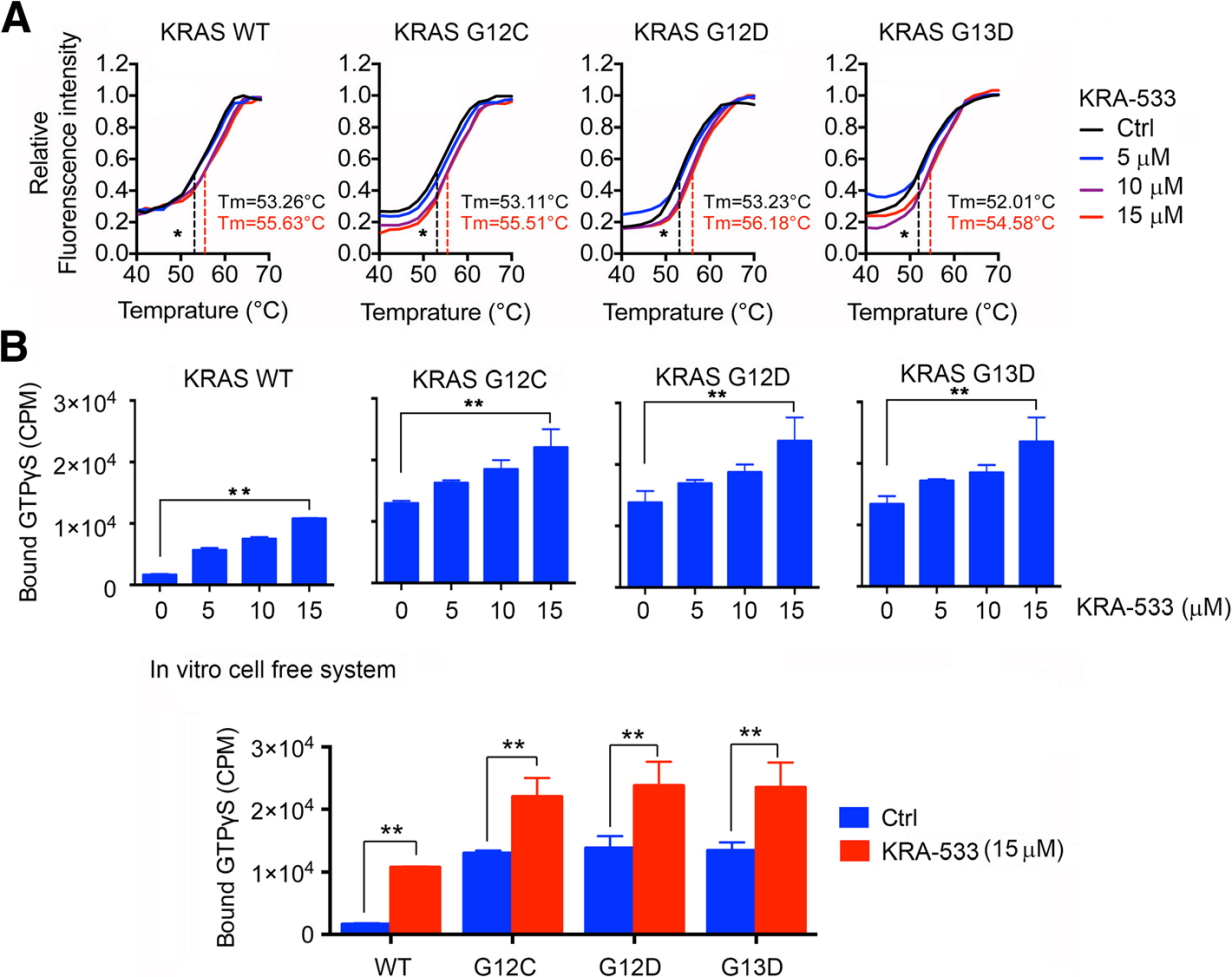
KRA-533 directly binds to and activates KRAS in an in vitro cell-free system. (**a**) Thermal shift melting curve of purified KRAS proteins (WT, G12C, G12D or G13D) incubated with increasing concentrations of KRA-533. Melting temperature (Tm) values of DMSO Ctrl and 15 μM of KRA-533 are shown. *P < 0.05 by 2-tailed *t* test. (**b**) 600 nM Purified KRAS protein (WT, G12C, G12D or G13D) was incubated with 11 μM [γ-^35^S] GTPγS, purified GFP (RASGRP1, 180 nM) and GAP (RASA1, 180 nM) at 25 °C in the presence or absence of increasing concentrations of KRA-533. The radioactivity remaining on the protein after intensive washing was quantified by liquid scintillation. Error bars represent ± SD. **P < 0.01 by 2-tailed *t* test

To test whether KRA-533 directly binds to KRAS, a thermal shift assay was employed as described [40]. Dose dependent increases in melting temperature (Tm) were observed when purified KRAS WT, G12C, G12D and G13D mutant proteins were incubated with increasing concentrations of KRA-533 (Fig. 2A). These results indicate that KRA-533 can directly bind to WT, G12C, G12D and G13D mutant KRAS proteins.

To further assess whether KRA-533 activates KRAS directly, an in vitro cell-free GDP-GTP exchange experiment was carried out as described [39]. Purified WT, G12C, G12D or G13D mutant KRAS proteins were incubated with purified GEF (RASGRP1) and GAP (RASA1) proteins in reaction buffer containing [γ-^35^S]-GTP in the absence or presence of increasing concentrations of KRA-533 at 25 °C for 60 min. KRAS activity was quantified by liquid scintillation counting. Mutant KRAS G12C, G12D and G13D displayed greater KRAS activity than WT KRAS in the absence of KRA-533 (Fig. 2B). Addition of KRA-533 activated WT KRAS to increase its activity in a dose-dependent manner. Intriguingly, KRA-533 further enhanced the activities of active KRAS mutants (i.e. G12C, G12D and G13D) (Fig. 2B). These findings indicate that KRA-533 not only activates WT KRAS but also has the capacity to further enhance KRAS activity of mutant KRAS.

### K117 is a required site for KRA-533 to bind and activate KRAS

**Fig. 3:**
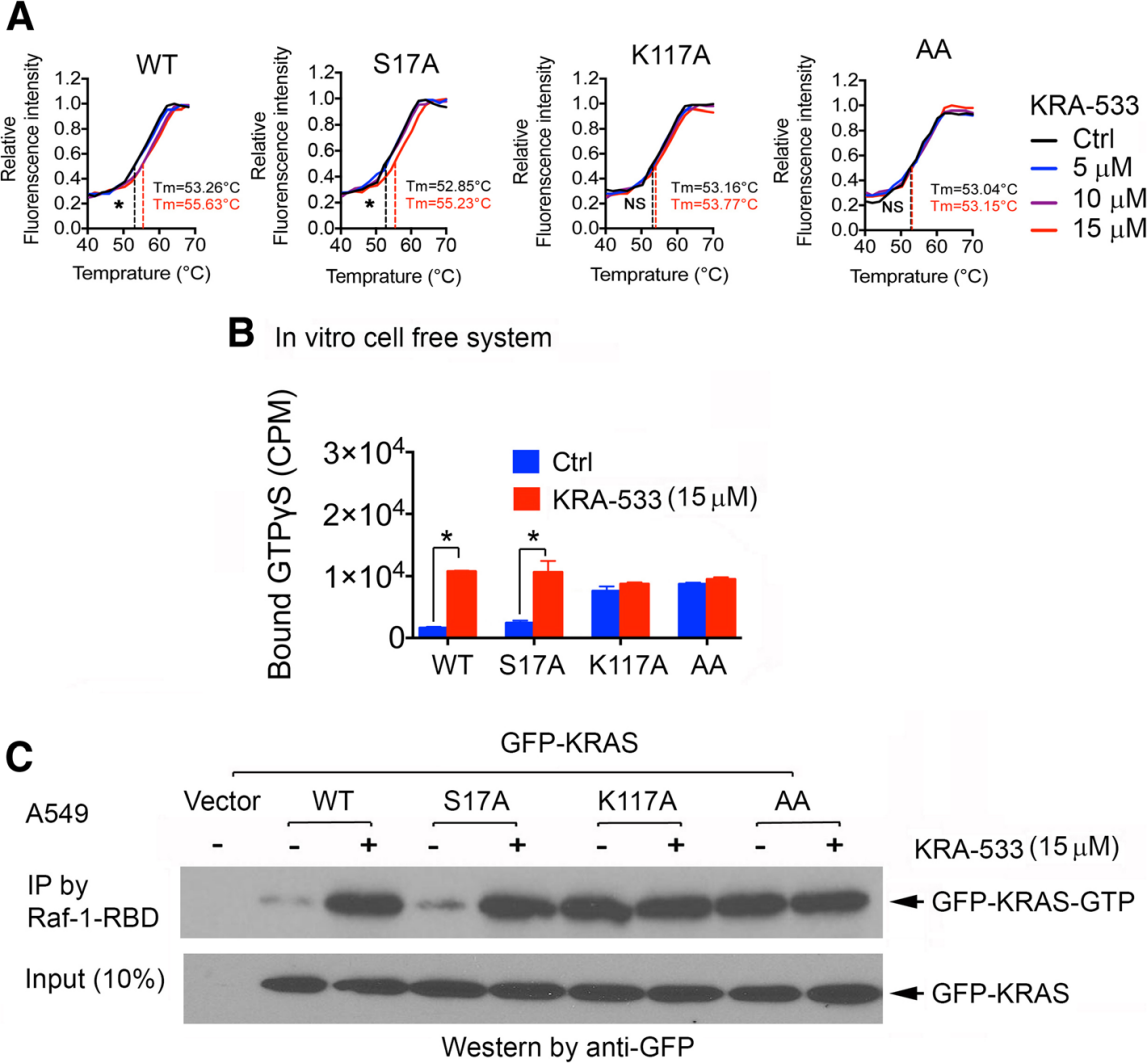
K117 site is essential for KRA-533 to bind and activate KRAS. (**a**) Thermal shift melting curve of purified KRAS protein (WT, S17A, K117A, or S17A/K117A (AA)) incubated with increasing concentrations of KRA-533. Melting temperature (Tm) values of DMSO control and 15 μM KRA-533 are shown. *P < 0.05, no significance → NS, by 2-tailed *t* test. (**b**) 600 nM purified KRAS protein (WT, S17A, K117A or AA) was incubated with 11 μM [γ-35S] GTPγS, purified GFP (RASGRP1, 180 nM) and GAP (RASA1, 180 nM) at 25 °C in the presence or absence of 15 μM KRA-533. The radioactivity remaining on the protein after intensive washing was quantified by liquid scintillation. Error bars represent ± SD. *P < 0.05 by 2-tailed *t* test. (**c**) A549 cells were transfected with GFP-tagged KRAS WT, S17A, K117A or AA, followed by treatment with KRA-533 (15 μM) for 48 h. KRAS·GTP (active KRAS) was pulled down by Raf-1-RBD. The GFP-tagged exogenous KRAS-GTP (GFP-KRAS-GTP) was analyzed by Western blot using anti-GFP antibody

Our findings reveal that KRA-533 not only binds to but also directly activates WT and most common KRAS mutants, including G12C, G12D and G13D. Structural computational modeling analysis reveals that KRA-533 is associated with 15 amino acids (Leu120, Asn85, Phe28, Glu31, Asp30, Ala18, Pro34, Val29, Asp33, Lys117, Val14, Lys16, Asp57**,** Ser17 and Tyr32) in the GDP/GTP binding pocket. Among KRA-533-associated amino acids in the KRAS protein, two hydrogen bonds are predicted with residues Ser17 and Lys117. We mutated these two sites to Ala individually or simultaneously, leading to generation of S17A, K117A and AA (i.e. S17A/K117A) KRAS mutants. Thermal shift assay indicates that KRA-533 could bind to recombinant WT and S17A KRAS proteins but failed to bind K117A and AA mutant KRAS proteins (Fig. 3A). Intriguingly, KRA-533 directly activated WT and S17A but not K117A mutant KRAS in a cell-free GDP-GTP exchange system (Fig. 3B). To further test this intracellularly, GFP-tagged WT, S17A, K117A and AA KRAS mutants were exogenously transfected into A549 cells. Then, cells were treated with KRA-533 for 48 h, followed by Raf-1-RBD beads pull-down. Activities of exogenous GFP-tagged WT and KRAS mutants were analyzed by Western blot using GFP antibody. Consistently, KRA-533 activated exogenous WT and S17A but not K117A KRAS mutant in A549 cells (Fig. 3C). These findings suggest that the Lys117 hydrogen-bond site is required for KRA-533 to bind or activate KRAS.

### KRA-533-induced KRAS activation promotes apoptosis and autophagic cell death in human lung cancer cells

**Fig. 4:**
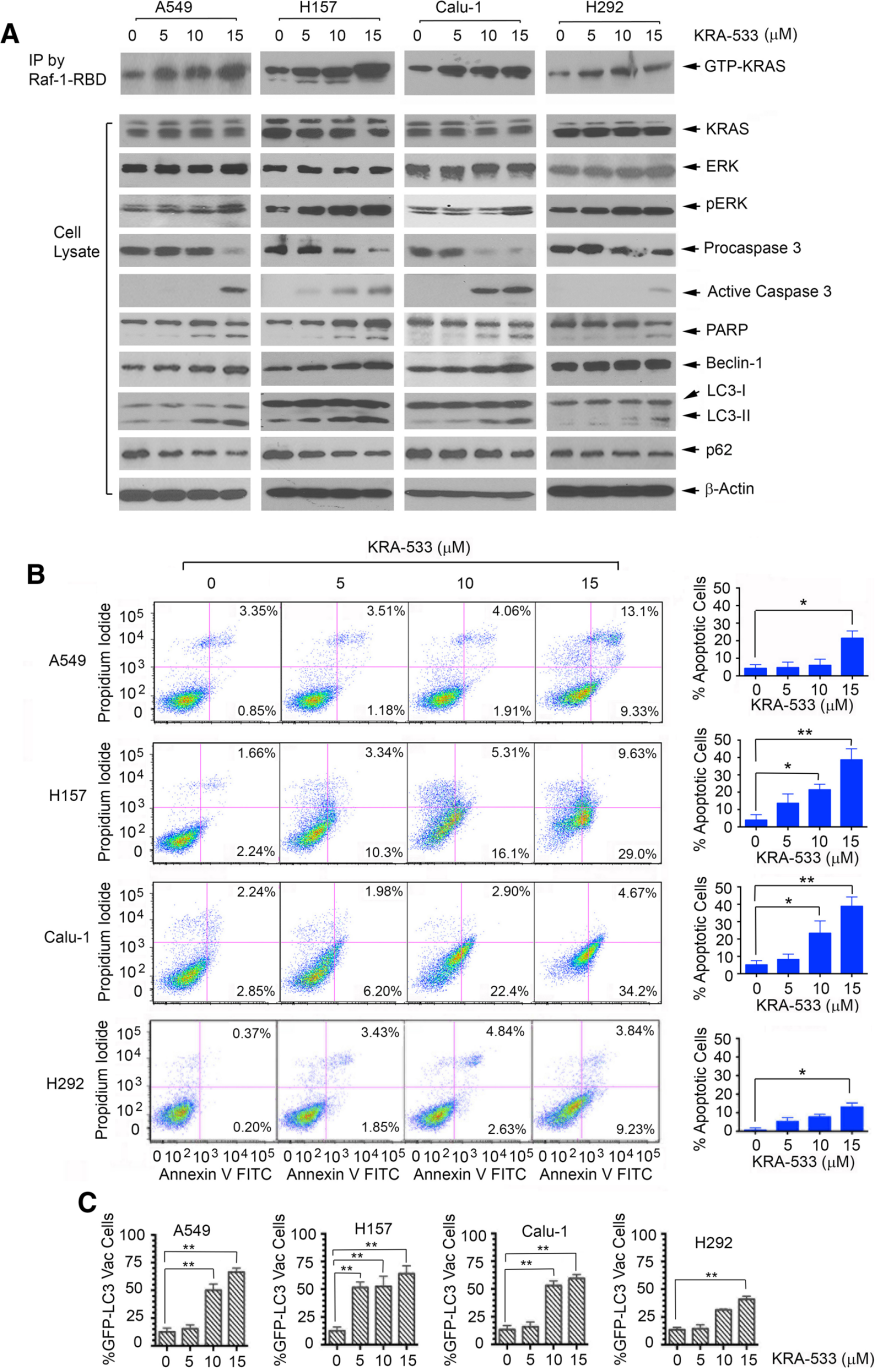
KRA-533 induces KRAS activation leading to apoptotic and autophagic cell death in human lung cancer cells. (**a**) and (**b**), A549, H157, Calu-1 and H292 cells were treated with increasing concentrations (0, 5, 10, 15 μM) of KRA-533 for 48 h. “0” means DMSO vehicle control. KRAS-GTP (active form of KRAS) was pulled down by Raf-1-RBD, followed by Western blot using KRAS antibody. Expression levels of KRAS, pERK, active caspase 3, PARP cleavage, Beclin1, LC3-I and LC3-II in total lysate were analyzed by Western blot. Apoptosis was determined by analyzing Annexin-V/PI binding by FACS. Data represent mean ± SD, *P < 0.05, **P < 0.01, by 2-tailed *t* test. (**c**) GFP-LC3 plasmids were transfected into A549, H157, Calu-1 and H292 cells. After 24 h, cells were treated with KRA-533 for 48 h. The percentages of LC3-GFP-transfected cells bearing LC3-GFP aggregates (LC3-FGPvac) were quantified as shown. Data represent mean ± SD, **P < 0.01, by 2-tailed *t* test

It has recently been reported that activated KRAS can trigger cell death via apoptosis and autophagy-associated cell death in cancer cells [1, 23–25]. To test whether KRA-533-activated KRAS promotes apoptosis and autophagic cell death, A549, H157, Calu-1 and H292 cells were treated with increasing concentrations of KRA-533 for 48 h, followed by analysis of KRAS activity, apoptosis and autophagy. KRA-533 enhanced KRAS activity in a dose-dependent manner, which was associated increased levels of pERK, ratio of active caspase 3/procaspase 3 and PARP cleavage, leading to apoptotic cell death determined by FACS analysis of Annexin V/PI staining (Fig. 4A and B**)**, measurement of caspase 3 activity using a Caspase 3 Colorimetric Assay Kit and mitochondrial membrane potential using JC-1 staining (Additional file 2**:** Figure S2). We also measured autophagy by analysis of LC3-I/LC3-II and p62 following treatment of cells with KRA-533. It is well known that p62 is an autophagy receptor or substrate that can be degraded by autophagy [52]. In addition to LC3-II, we analyzed p62 as another autophagy marker. Intriguingly, KRA-533 induced a dose-dependent increase of LC3-II and a dose-dependent decrease of p62 in A549, H157, Calu-1 cells and H292 (Fig. 4A). To further quantify the level of autophagy, a GFP-LC3 construct was used to indicate autophagosomes as previously described [49, 53]. After treatment with KRA-533, GFP-LC3 redistributed from a diffuse staining pattern in the cytoplasm and nucleus to a cytoplasmic punctate structure that specifically labels pre-autophagosomal and autophagosomal membranes (i.e. GFP-LC3 vac cells, Additional file 3**:** Figure S3). Intriguingly, KRA-533 enhanced the percentage of GFP-LC3vac cells in a dose-dependent manner (Fig. 4C). These findings indicate that, in addition to apoptosis, KRA-533 can also induce autophagic cell death. Importantly, A549, H157 and Calu-1 cells with KRAS mutation were significantly more sensitive than H292 cells without KRAS mutation to KRA-533-stimulated KRAS activation, induction of apoptosis and autophagy (Fig. 4), suggesting that KRA-533 may be relatively selective for cancer cells bearing KRAS mutation(s).

To assess whether the autophagy inhibitor regulates KRA-533-induced autophagy formation, A549 and H157 cells were treated with KRA-533 in the absence or presence of autophagy inhibitor chloroquine for 48 h, followed by Western blot analysis of the autophagy marker p62. KRA-533 induced autophagy with deceased p62 level via degradation. The autophagy inhibitor chloroquine restored p62 expression by blocking KRA-533-induced p62 degradation. These findings indicate that autophagy inhibitor chloroquine has ability to block KRA-533-induced autophagy (Additional file 4**:** Figure S4).

To determine whether autophagy plays a role in apoptosis induced by KRA-533, we measured apoptosis by FACS analysis of Annexin V/PI staining following treatments of A549 and H157 with KRA533 alone or in combination with autophagy inhibitor 3-MA for 48 h. Results show that the autophagy inhibitor 3-MA enhanced KRA-533-induced apoptosis (Additional file 5**:** Figure S5).

To further test whether KRAS is a required target for KRA-533 to induce apoptotic and autophagic cell death, G12S mutant KRAS was depleted using KRAS shRNA from A549 cells, followed by treatment with KRA-533 (15 μM) for 48 h. Knockdown of mutant KRAS significantly reduced cell sensitivity to the induction of apoptotic and autophagic cell death by KRA-533 (Additional file 6**:** Figure S6). These findings suggest that KRAS may be an essential target for KRA-533 against lung cancer via apoptotic and autophagic cell death pathways.

### KRA-533 suppresses mutant KRAS lung cancer in xenograft models

To define the appropriate doses of KRA-533 for in vivo experimentation, we first determined standard single-dose maximum tolerated dose (MTD) as previously described [49]. Nu/Nu nude mice were treated with a single dose of 150, 300 or 400 mg/kg i.p., followed by toxicity observations. Treatment of mice with a single dose of 150 or 300 mg/kg i.p. did not cause weight loss or other toxicities, including hematologic disorders, or liver and kidney function abnormalities (Additional file 7**:** Figure S7). However, a single dose of 400 mg/kg resulted in death of mice in 8 days. Alanine transaminase (ALT), aspartate transaminase (AST) and blood urea nitrogen (BUN) were significantly elevated (Additional file 7**:** Figure S7). Based on these findings, mice might die mainly from liver and kidney damage at a single 400 mg/kg dose. Thus, the single dose MTD of KRA-533 ranges from 300 to 400 mg/kg. 10% of single-dose MTD can usually be considered the maximum therapeutic dose (~ 30–40 mg/kg) for continuous treatment. We therefore considered doses between 10 and 30 mg/kg/day to be relatively safe.

**Fig. 5:**
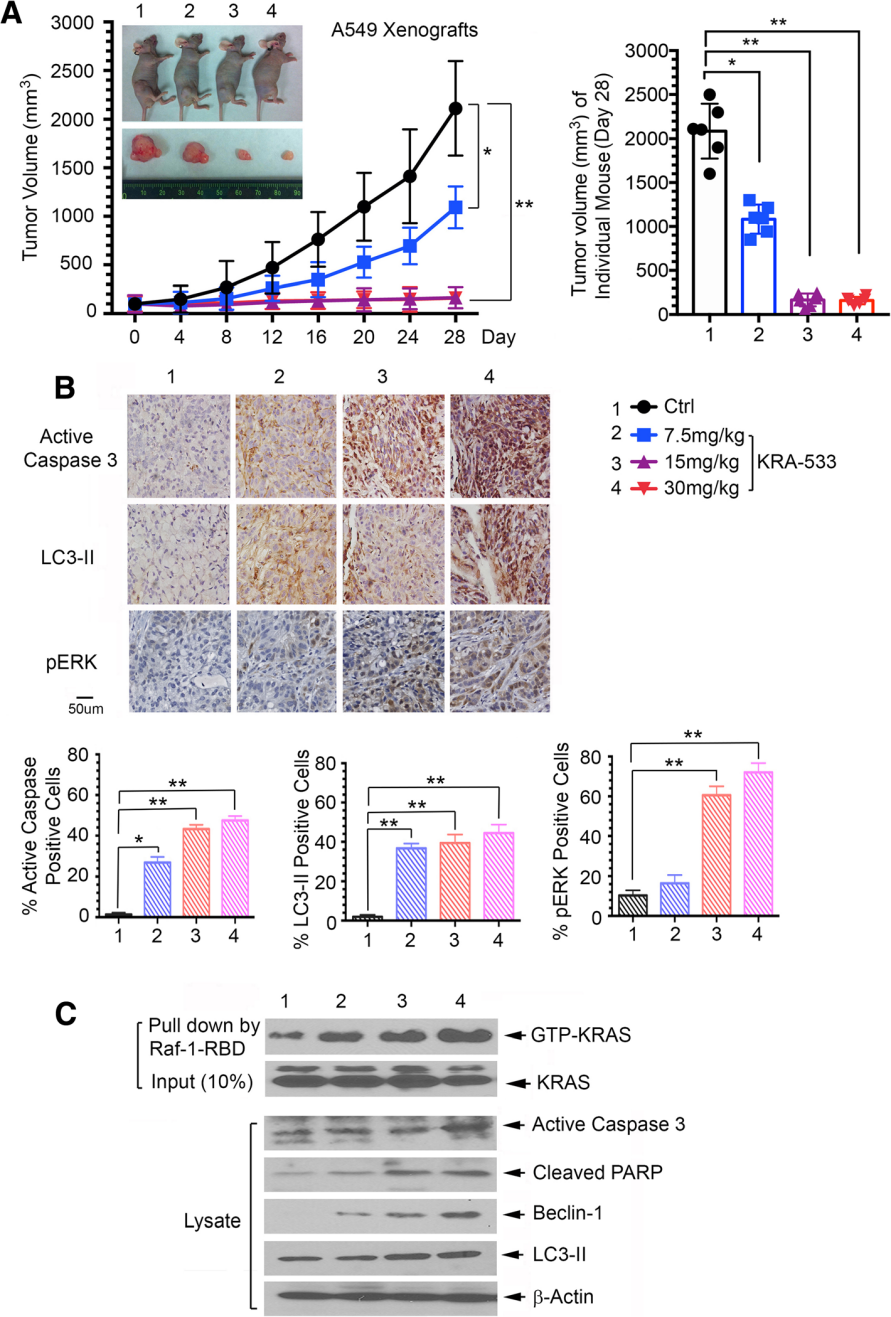
KRA-533 potently suppresses tumor growth in KRAS mutant lung cancer xenografts. (**a**) Nu/Nu nude mice with A549 xenografts bearing mutant KRAS were treated with increasing doses of KRA-533 (0, 7.5, 15, and 30 mg/kg/d) for 28 days (*n* = 6). Tumor volume was measured once every 2 days (left panel). Tumor volumes of 6 individual mice in each group were compared on day 28 (right panel). After 28 days, the mice were sacrificed, and the tumors were removed and analyzed. Data represent mean ± SD, n = 6 per group. *P < 0.05, **P < 0.01, by 2-tailed *t* test. (**b**) Active caspase-3, LC3-II and p-ERK were analyzed by IHC staining in tumor tissues at the end of experiments and quantified. Data represent mean ± SD, n = 6 per group. *P < 0.05, **P < 0.01, by 2-tailed *t* test. (**b**) KRAS-GTP (active form of KRAS) was pulled down by Raf-1-RBD from tumor tissue lysates, followed by Western blot using KRAS antibody. Expression levels of active caspase-3, cleaved PARP, Beclin-1 and LC3-II in tumor tissues were analyzed by Western blot

To test the potency of KRA-533 in vivo, lung cancer xenografts derived from A549 cells bearing KRAS mutation (G12S) were treated with increasing doses (0, 7.5, 15, and 30 mg/kg/day) of KRA-533 i.p. for 28 days. KRA-533 suppressed tumor growth in a dose-dependent manner in lung cancer mutant KRAS xenografts (Fig. 5A). To assess whether KRA-533 induced suppression of tumor growth via apoptosis and autophagy in vivo, representative samples from harvested tumor tissues were analyzed by immunohistochemistry (IHC) for active caspase-3 or LC3-II as described [45, 46]. Indeed, KRA-533 induced apoptosis and autophagy in tumor tissues in a dose-dependent manner (Fig. 5B). Raf-1-RBD pull-down experiments for KRAS activity were also carried out using total cell lysates isolated from tumor tissues. Treatment of mice with KRA-533 resulted in accumulation of active KRAS in tumor tissues in association with increased apoptosis and autophagy (Fig. 5C), suggesting KRA-533-mediated tumor suppression may occur through induction of apoptosis and autophagic cell death.

**Fig. 6:**
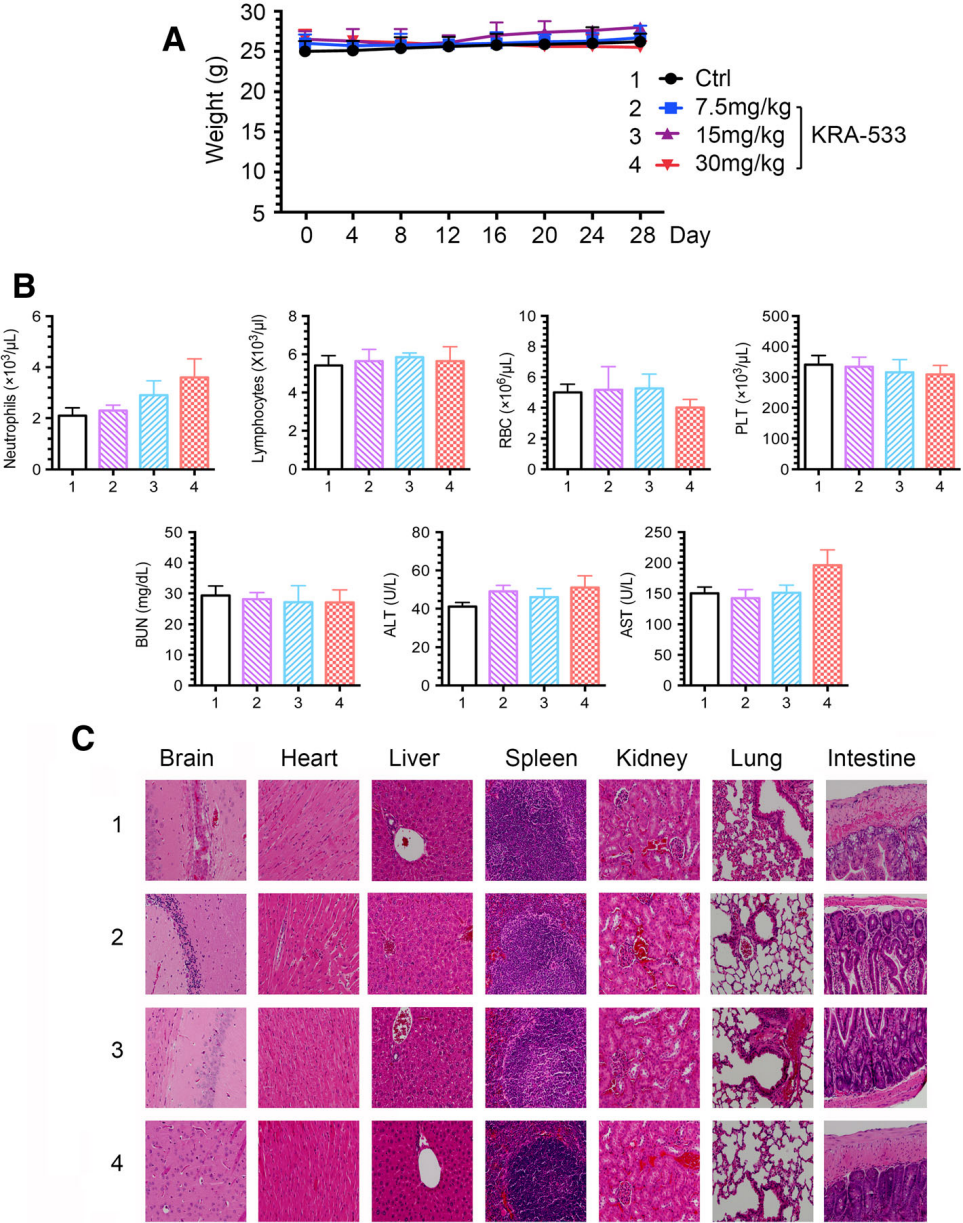
Toxicity of KRA-533 in vivo. (**a**), (**b**) and (**c**) Body weight, blood analysis and H&E histology of various organs from mice bearing A549 xenografts after treatment with various doses of KRA-533 for 28 days

Treatment was well tolerated without significant toxicity within effective dose range (7.5 ~ 30 mg/kg/d). There was no weight loss (Fig. 6A). Tests of blood cells (WBC, RBC and PLT) for bone marrow, BUN for kidney and ALT/AST for liver functions were in the normal range (Fig. 6B). Histopathology of harvested normal tissues (brain, heart, lung, liver, spleen, kidney and intestine) revealed no evidence of normal tissue toxicities after treatment with doses of 7.5~30 mg/kg/day (Fig. 6C). These findings suggest that doses between 7.5 and 30 mg/kg provide the optimal therapeutic index for KRA-533 for in vivo studies.

### KRA-533 potently inhibits tumor growth and prolongs survival of mice with genetically engineered G12D mutant KRAS-driven lung cancer

**Fig. 7:**
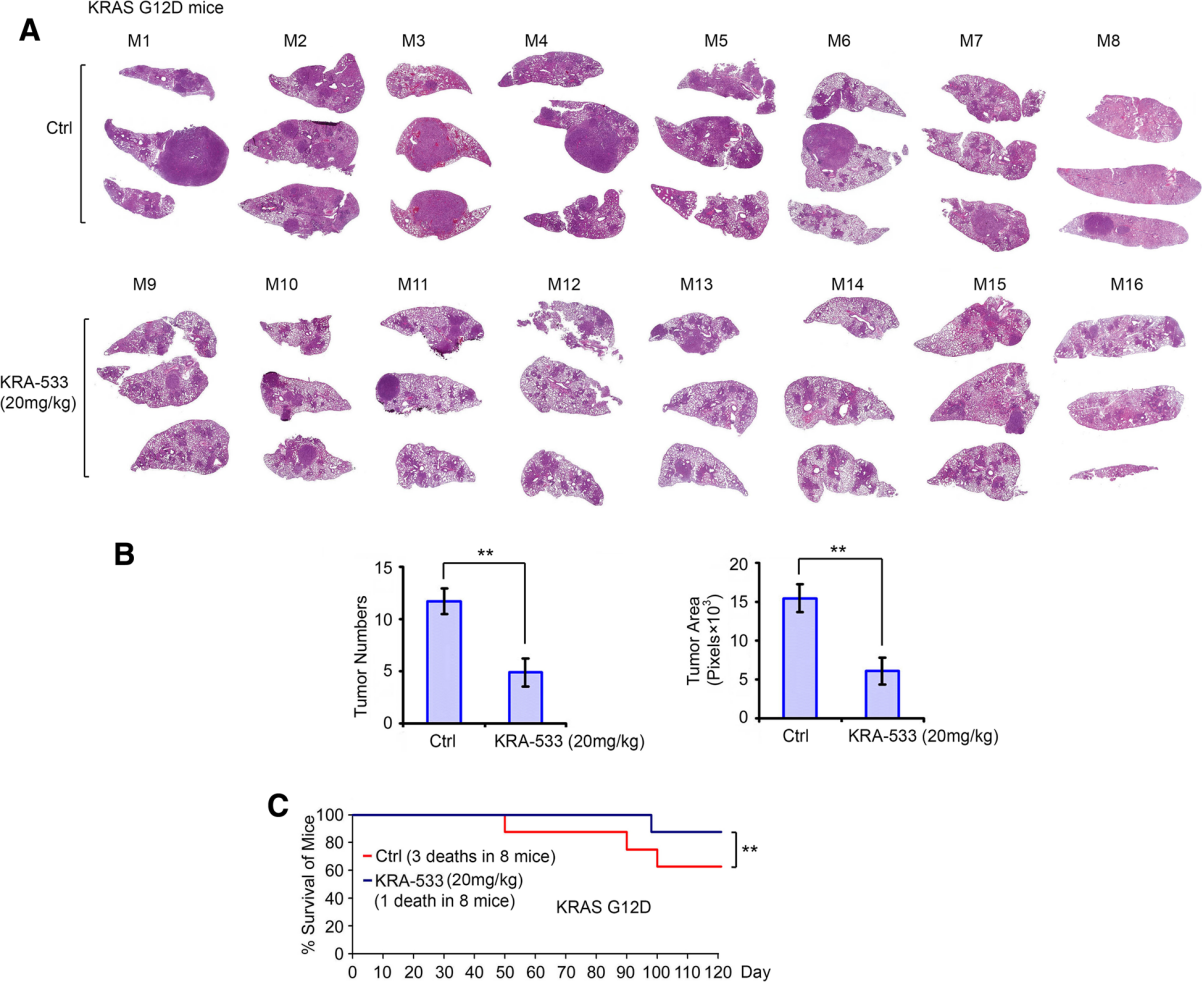
KRA-533 inhibits mutant KRAS^G12D^ driven lung cancer growth in genetically engineered mouse model. (**a**) After administration of adenovirus Cre recombinase in KRAS^G12D^ mice for 10 weeks, mice were treated with KRA-533 (20 mg/kg/d) for four months (*n* = 8 each group). H&E images from control or treatment group are shown. (**b**) Tumor numbers were counted under the microscope and tumor area was quantified using Openlab modular imaging software. Data represent mean ± SD, n = 8 per group. **P < 0.01, by 2-tailed *t* test. (**c**) Survival of mice was calculated up to four months before euthanization in the control group versus the KRA-533 treatment group. Data represent mean ± SD, n = 8 per group. **P < 0.01, by 2-tailed *t* test

KRAS mutations are common genetic alterations in NSCLC and contribute to the resistance of lung cancer to conventional therapy [11, 12, 18]. To test the potency of KRA-533 in mutant KRAS-driven lung cancer, we generated lox-stop-lox (LSL*)*-KRAS^G12D^ mice as previously described [47, 48, 54, 55]. By engineering LoxP DNA elements into the mouse genome that surround a synthetic ‘stop’ element (lox-stop-lox) inserted in front of mutant KRAS G12D, we can ‘turn-on’ mutant KRAS G12D with delivery of Cre recombinase [56]. To control the expression of KRAS G12D, a LSL cassette was engineered into the first intron of the KRAS gene. The LSL cassette consists of transcriptional and translational stop elements flanked by LoxP sites that prevent the expression of the mutant allele until the stop elements are removed by the activity of Cre recombinase [54, 55]. To produce KRAS G12D mutant-driven lung cancer, alleles were induced in mouse lung using intranasal administration of a lentiviral Cre recombinase. Primary lung tumors developed around 12 weeks post-inoculation. To assess whether KRA-533 has antitumor activity against G12D mutant KRAS-driven lung cancer in genetically engineered mouse models, KRA-533 (20 mg/kg/d) or vehicle control was administered i.p. starting at 10 weeks post AdeCre delivery as previously suggested [48]. After treatment for 3 months, mice were euthanized for analysis of tumor burden as previously described [46, 48]. Treatment of KRAS G12D mice with KRA-533 for four months resulted in significant reduction of tumor burden and multiplicity in the lung (Fig. 7A and B). Importantly, KRA-533 prolonged survival of KRAS G12D mice compared with the control group (Fig. 7C). There were 3 deaths out of 8 mice in the control group versus 1 death out of 8 mice in the KRA-533 treatment group (*p* < 0.01) in four months before euthanization. Slight weight loss but no significant normal tissue toxicities were observed in mice (Additional file 8**:** Figure S8).

Around half of KRAS-mutant lung cancer patients also carry LKB1 mutations, therefore, it will be important to test whether KRA-533 is also effective for the treatment of KRAS-mutant lung cancers with LKB1 mutation or loss. LSL-KRAS^G12D^ LKB1^fl/fl^ (KL) mice were generated by intercrossing B6.129S4-Krastm3Tyj/J (KRAS^G12D^) and FVB; 129S6-Stk11tm1Rdp/Nci (LKB1) strains two generations and genotyped to confirm homozygosity for the LKB1 allele. These mice contain a KRAS^G12D^ LSL knock-in allele and a floxed allele of LKB1 (LKB1^fl/fl^) [47, 48]. KRA-533 (20 mg/kg/d) or vehicle control was administered i.p. starting at 6 weeks post AdeCre delivery as previously suggested [46, 48]. After treatment for 8 weeks, mice were euthanized and lung tumors were analyzed as described above. Results reveal that KRA-533 also significantly suppressed tumor growth and prolonged survival of KL mice (Additional file 9**:** Figure S9).

## Discussion

Small-molecule drugs act by binding to proteins; those proteins that harbor sites amenable to small molecule binding are termed “druggable” [6, 57]. Despite more than three decades of intensive effort, no effective pharmacologically direct inhibitors of the RAS oncoproteins have reached the clinic, prompting the widely held perception that RAS proteins are ‘undruggable’ [5]. Creation of therapeutic agents that directly inhibit the oncogenic effects of RAS proteins has been challenging [5]. Recently, direct KRAS G12C specific inhibitors have been discovered, which irreversibly bind to a common oncogenic mutant, KRAS (G12C) and allosterically control GTP affinity and effector interactions [38]. Based on this finding, a more potent compound, ARS-853, has been developed as an allele-specific inhibitor to selectively target and inactivate mutant KRAS G12C [58, 59]. ARS-853 binds to the switch II pocket of GDP-bound KRAS-G12C, and leads to a generally inactive state of KRAS. KRAS-G12C is able to cycle between GDP- and GTP-bound states while ARS-853 inhibits this cycling. KRAS G12C is unable to achieve the GTP-bound state once bound by ARS-853 [58]. ARS-853 potently suppresses the growth of cancer cells bearing G12C mutant KRAS, while cancer cells that express non-G12C mutants (i.e. G12D, G12S, G12 V, G13D, Q61K, etc.) are resistant to ARS-853 [58]. In addition to ARS-853, a small-molecule pan-RAS inhibitor (compound 3144), was recently discovered by targeting multiple adjacent sites on KRAS G12D protein near D38, A59 and Y32 sites [6]. Compound 3144 can prevent effector protein binding and causes tumor growth inhibition in animal models of RAS dependent cancers [6].

RAS has opposing functions in cell proliferation/survival versus cell death pathways [1, 23–25]. Here we used the GTP/GDP-binding pocket of KRAS as a docking site to screen the NCI library of small molecules using the UCSF-DOCK program suite. KRA-533 was identified as a lead compound that targets the GTP/GDP-binding pocket in KRAS protein. KRA-533 potently enhances intracellular KRAS activities to various degrees in a series of human NSCLC cell lines in association with various degrees of growth inhibition. Intriguingly, lung cancer cell lines bearing KRAS mutation are more sensitive to KRA-533 than those without KRAS mutation, indicating KRA-533 may be relatively selective for mutant KRAS lung cancer cells. KRA-533 not only directly binds to WT KRAS protein but also KRAS mutants, including G12C, G12D and G13D. Although KRAS mutants have higher levels of activities than WT KRAS before KRA-533 treatment, KRA-533 induces a dose-dependent increase in activity of WT and mutant KRAS in cell-free GDP-GTP exchange assay and in lung cancer cells. Compared to WT KRAS, the G12C, G12D and G13D KRAS mutants become hyperactive following KRA-533 treatment. It has been reported that glucose withdrawal or glucose-mediated hyperactivation of RAS is able to trigger apoptosis [26, 28]. Hyperactivation of RAS can also trigger autophagy-associated cell death [1]. Here we found that KRA-533-induced hyperactivation of mutant KRAS led to apoptosis and autophagic cell death in mutant KRAS lung cancer cell lines (i.e. A549, H157 and Calu-1). In contrast, the moderate level of WT KRAS activation induced by KRA-533 caused significantly less apoptosis and autophagic cell death in a lung cancer cell line without KRAS mutation (i.e. H292). Therefore, there may be a threshold of KRAS activity in cells to dictate pathway choice between survival and death. Based our findings and previous reports, we propose that KRAS activity at or below the threshold may promote cell survival and proliferation while KRAS activity beyond the threshold (i.e. hyperactivation or super-activity) may promote cell death. KRA-533 may change the nature of KRAS signaling from pro-survival to pro-death by stimulating active mutant KRAS to a super-active status that is beyond the activity threshold.

Our data indicate that KRA-533 not only binds KRAS but also directly activates its activity. Structural modeling analysis by computational programming reveals that KRA-533 is associated with 15 amino acids in the GDP/GTP binding pocket, including the hydrogen-bond predicted with residue K117. Mutation of K117 to Ala resulted in KRAS loss of KRA-533 binding capacity. KRA-533 failed to activate K117A mutant KRAS. These findings indicate that, in addition to GTP binding [60], the K117 site is also critical for KRA-533 to bind to and/or activate KRAS. Intriguingly, a mutation of K117 in KRAS (K117 N) has been found in cancer patients [21, 22], indicating that K117 should be an important site in KRAS for regulation of its activity.

KRA-533 exhibited potent antitumor activity against mutant KRAS lung cancer via induction of KRAS hyperactivation, apoptosis and autophagic cell death in NSCLC xenografts. Importantly, the dose range between 10 and 30 mg/kg/day was effective without significant normal tissue toxicity in murine lung cancer models. Genetically engineered mice are the most sophisticated animal models of human cancer, which closely recapitulate the pathophysiological process of human malignancies in genetically precisely defined systems [56]. Therefore, the potency of KRA-533 was evaluated in genetically engineered LSL-KRAS^G12D^ and LSL-KRAS^G12D^ LKB1^fl/fl^ (KL) mice. Importantly, KRA-533 significantly reduces tumor burden in the lungs of both LSL-KRAS^G12D^ and KL mice, leading to prolonged survival compared to the untreated control group, suggesting that KRA-533 has potential to improve the prognosis of mutant KRAS driven lung cancer.

## Conclusion

We have discovered KRA-533 as a new class of KRAS agonist that selectively targets the GTP/GDP binding pocket. The binding of KRA-533 with KRAS promotes accumulation of GTP-KRAS probably by prevention of cleavage from GTP into GDP. KRA-553-induced hyperactivation of mutant KRAS facilitates apoptotic and autophagic cell death in mutant KRAS lung cancer cells. KRA-533 also displays potent efficacy against tumor growth in mutant KRAS xenografts and genetically engineered mutant KRAS driven lung cancer. Development of this KRAS agonist may offer an effective approach for the treatment of mutant KRAS lung cancer.

## Additional files

Additional file 1: **Figure S1.** KRA-533 inhibits proliferation of NSCLC cells. (A) A549, H157, Calu-1 and H292 cells were treated with increasing concentrations of KRA-533, followed by analysis of cell proliferation using MTS Cell Proliferation Colorimetric Assay Kit. (B) IC50 values of KRA533 based on cell proliferation data from (A). (JPG 700 kb)

Additional file 2: **Figure S2.** KRA-533 induces caspase 3 activation and reduces mitochondrial membrane potential in NSCLC cells. (A) and (B) A549, H157, Calu-1 and H292 cells were treated with increasing concentrations of KRA-533, followed by analysis of caspase 3 activity using Caspase 3 Colorimetric Assay Kit (A) and measurement of mitochondrial membrane potential by JC-1 staining (B). Data represent mean ± SD, *P < 0.05, **P < 0.01, by 2-tailed *t* test. (JPG 2498 kb)

Additional file 3: **Figure S3.** KRA-533 induces autophagy formation in NSCLC cells. A549, H157, Calu-1 and H292 cells were transfected with *GFP-LC3*. After 24 h, cells were treated with KRA-533 for 48 h. Autophagic vacuoles in the representative cells from various treatments were shown. Scale bar represents 20 μm. (JPG 354 kb)

Additional file 4: **Figure S4.** The autophagy inhibitor chloroquine blocks KRA-533-induced autophagy in NSCLC cells. A549 and H157 cells were treated with KRA-533 (10 μM) in the absence or presence of autophagy inhibitor chloroquine (10 μM) for 48 h, followed by Western blot analysis of the autophagy marker p62. (JPG 187 kb)

Additional file 5: **Figure S5.** The autophagy inhibitor 3-methyladenine (3-MA) enhanced KRA-533-induced apoptosis of NSCLC cells. A549 and H159 cells were treated with KRA-533 (10 μM) in the absence or presence of 3-MA for 48 h, followed by FACS analysis of Annexin V/PI staining for apoptosis. Data represent mean ± SD, *P < 0.05, by 2-tailed *t* test. (JPG 362 kb)

Additional file 6: **Figure S6.** Silencing of mutant KRAS reduced sensitivity of cells to KRA-533. (A) KRAS shRNA plasmids were transfected into A549 cells that contain KRAS mutation, followed by Western blot using KRAS antibody. (B) A549 cells and mutant KRAS silenced A549 cells were treated with KRA-533 (15 μM) for 48 h. Apoptotic cells were detected by Annexin V /PI binding and analyzed by FACS. Data represent mean ± SD, **P < 0.01, by 2-tailed *t* test. (C) GFP-LC3 constructs and KRAS shRNA plasmids were co-transfected into A549 cells, followed by treatment with KRA-533 for 48 h. Autophagic cells (GFP-LC3 positive cells) were visualized by Axioplan Zeiss microscope and quantified. Data represent mean ± SD, **P < 0.01, by 2-tailed *t* test. (JPG 653 kb)

Additional file 7: **Figure S7.** Determination of single dose maximum tolerated dose (MTD). (A) Nu/Nu nude mice were treated with single dose (i.e. 0, 150 mg/kg, 300 mg/kg or 400 mg/kg) KRA-533 via i.p. (*n* = 6 mice per group). After treatment, the body weight of mice was measured once every other day for 2 weeks. (B) Blood analysis of mice after treatment with single dose(s) of KRA-533. (C) H&E histology of various organs from mice after treatment with single dose(s) of KRA-533. (JPG 1446 kb)

Additional file 8: **Figure S8.** Toxicity of KRA-533 in genetically engineered LSL-KRAS ^G12D^ mice. (A), (B) and (C), Body weight, blood analysis and H&E histology of various organs from mice bearing A549 xenografts after treatment with KRA-533 (20 mg/kg/d) for 4 months. (JPG 2133 kb)

Additional file 9: **Figure S9.** Potency of KRA-533 in genetically engineered LSL-KRAS^G12D^ LKB1^fl/fl^ (KL) mice. (A) and (B) After administration of adenovirus Cre recombinase in KL mice for 6 weeks, mice were treated with KRA-533 (20 mg/kg/d) for 8 weeks (n = 6 each group). Three representative brightfield images of mouse lungs and H&E images from control or KRA-533 treatment group are shown. (C) Tumor numbers were counted under the microscope and tumor area was quantified using Openlab modular imaging software. Data represent the mean ± SD, n = 6 per group. **P < 0.01, by 2-tailed *t* test. (D) Survival of mice was calculated up to 8 weeks before euthanization in the control group versus the KRA-533 treatment group. Data represent mean ± SD, n = 6 per group. **P < 0.01, by 2-tailed *t* test. (JPG 3111 kb)

## Abbreviations

ALT: Alanine aminotransferase
AST: Aspartate aminotransferase
BUN: Blood urea nitrogen
KRA-533: KRAS agonist-533
KRAS: Kirsten rat sarcoma viral oncogene
MTD: Maximum tolerated dose
NSCLC: Non-small cell lung cancer
SRB: Sulforhodamine B

## References

[1] Overmeyer JH, Maltese WA (2011). Death pathways triggered by activated Ras in cancer cells. Front Biosci (Landmark Ed).

[2] Hancock JF (2003). Ras proteins: different signals from different locations. Nat Rev Mol Cell Biol.

[3] Downward J (2003). Targeting RAS signalling pathways in cancer therapy. Nat Rev Cancer.

[4] Stephen AG, Esposito D, Bagni RK, McCormick F (2014). Dragging ras back in the ring. Cancer Cell.

[5] Cox AD, Fesik SW, Kimmelman AC, Luo J, Der CJ (2014). Drugging the undruggable RAS: Mission possible?. Nat Rev Drug Discov.

[6] Welsch ME, Kaplan A, Chambers JM, Stokes ME, Bos PH, Zask A, Zhang Y, Sanchez-Martin M, Badgley MA, Huang CS (2017). Multivalent small-molecule pan-RAS inhibitors. Cell.

[7] Hall BE, Bar-Sagi D, Nassar N (2002). The structural basis for the transition from Ras-GTP to Ras-GDP. Proc Natl Acad Sci U S A.

[8] Scheele JS, Rhee JM, Boss GR (1995). Determination of absolute amounts of GDP and GTP bound to Ras in mammalian cells: comparison of parental and Ras-overproducing NIH 3T3 fibroblasts. Proc Natl Acad Sci U S A.

[9] Affolter A, Drigotas M, Fruth K, Schmidtmann I, Brochhausen C, Mann WJ, Brieger J. Increased radioresistance via G12S K-Ras by compensatory upregulation of MAPK and PI3K pathways in epithelial cancer. Head Neck. 2012. 10.1002/hed.22954 22302684

[10] Okudela K, Hayashi H, Ito T, Yazawa T, Suzuki T, Nakane Y, Sato H, Ishi H, KeQin X, Masuda A (2004). K-ras gene mutation enhances motility of immortalized airway cells and lung adenocarcinoma cells via Akt activation**:** possible contribution to non-invasive expansion of lung adenocarcinoma. Am J Pathol.

[11] Okudela K, Woo T, Kitamura H (2010). KRAS gene mutations in lung cancer: particulars established and issues unresolved. Pathol Int.

[12] Ding L, Getz G, Wheeler DA, Mardis ER, McLellan MD, Cibulskis K, Sougnez C, Greulich H, Muzny DM, Morgan MB (2008). Somatic mutations affect key pathways in lung adenocarcinoma. Nature.

[13] Park MJ, Park SH, Park PW, Seo YH, Kim KH, Jeong JH, Kim MJ, Ahn JY, Lee JH, Park J, Hong J (2013). Frequency of KRAS mutations in adult Korean patients with acute myeloid leukemia. Int J Hematol.

[14] Al-Kzayer LF, Sakashita K, Al-Jadiry MF, Al-Hadad SA, Ghali HH, Uyen le TN, Liu T, Matsuda K, Abdulkadhim JM, Al-Shujairi TA (2015). Analysis of KRAS and NRAS gene mutations in Arab Asian children with acute leukemia: high frequency of RAS mutations in acute lymphoblastic leukemia. Pediatr Blood Cancer.

[15] Burmer GC, Loeb LA (1989). Mutations in the KRAS2 oncogene during progressive stages of human colon carcinoma. Proc Natl Acad Sci U S A.

[16] Almoguera C, Shibata D, Forrester K, Martin J, Arnheim N, Perucho M (1988). Most human carcinomas of the exocrine pancreas contain mutant c-K-ras genes. Cell.

[17] Tam IY, Chung LP, Suen WS, Wang E, Wong MC, Ho KK, Lam WK, Chiu SW, Girard L, Minna JD (2006). Distinct epidermal growth factor receptor and KRAS mutation patterns in non-small cell lung cancer patients with different tobacco exposure and clinicopathologic features. Clin Cancer Res.

[18] Sanders HR, Albitar M (2010). Somatic mutations of signaling genes in non-small-cell lung cancer. Cancer Genet Cytogenet.

[19] Meng D, Yuan M, Li X, Chen L, Yang J, Zhao X, Ma W, Xin J (2013). Prognostic value of K-RAS mutations in patients with non-small cell lung cancer: a systematic review with meta-analysis. Lung Cancer.

[20] Riely GJ, Marks J, Pao W (2009). KRAS mutations in non-small cell lung cancer. Proc Am Thorac Soc.

[21] Janakiraman M, Vakiani E, Zeng Z, Pratilas CA, Taylor BS, Chitale D, Halilovic E, Wilson M, Huberman K, Ricarte Filho JC (2010). Genomic and biological characterization of exon 4 KRAS mutations in human cancer. Cancer Res.

[22] Stolze B, Reinhart S, Bulllinger L, Frohling S, Scholl C (2015). Comparative analysis of KRAS codon 12, 13, 18, 61, and 117 mutations using human MCF10A isogenic cell lines. Sci Rep.

[23] Overmeyer JH, Kaul A, Johnson EE, Maltese WA (2008). Active ras triggers death in glioblastoma cells through hyperstimulation of macropinocytosis. Mol Cancer Res.

[24] Bhanot H, Young AM, Overmeyer JH, Maltese WA (2010). Induction of nonapoptotic cell death by activated Ras requires inverse regulation of Rac1 and Arf6. Mol Cancer Res.

[25] Khokhlatchev A, Rabizadeh S, Xavier R, Nedwidek M, Chen T, Zhang XF, Seed B, Avruch J (2002). Identification of a novel Ras-regulated proapoptotic pathway. Curr Biol.

[26] Peeters K, Van Leemputte F, Fischer B, Bonini BM, Quezada H, Tsytlonok M, Haesen D, Vanthienen W, Bernardes N, Gonzalez-Blas CB (2017). Fructose-1,6-bisphosphate couples glycolytic flux to activation of Ras. Nat Commun.

[27] Yun J, Mullarky E, Lu C, Bosch KN, Kavalier A, Rivera K, Roper J, Chio II, Giannopoulou EG, Rago C (2015). Vitamin C selectively kills KRAS and BRAF mutant colorectal cancer cells by targeting GAPDH. Science.

[28] Telang S, Yalcin A, Clem AL, Bucala R, Lane AN, Eaton JW, Chesney J (2006). Ras transformation requires metabolic control by 6-phosphofructo-2-kinase. Oncogene.

[29] Corcoran RB, Cheng KA, Hata AN, Faber AC, Ebi H, Coffee EM, Greninger P, Brown RD, Godfrey JT, Cohoon TJ (2013). Synthetic lethal interaction of combined BCL-XL and MEK inhibition promotes tumor regressions in KRAS mutant cancer models. Cancer Cell.

[30] Zhang J, Park D, Shin DM, Deng X (2016). Targeting KRAS-mutant non-small cell lung cancer: challenges and opportunities. Acta Biochim Biophys Sin Shanghai.

[31] Ye N, Zhou J. KRAS - an evolving Cancer target. Austin J Cancer Clin Res. 2014:1. PMC5026231 27642639

[32] Spiegel J, Cromm PM, Zimmermann G, Grossmann TN, Waldmann H (2014). Small-molecule modulation of Ras signaling. Nat Chem Biol.

[33] Ball ED, Sorenson GD, Pettengill OS (1986). Expression of myeloid and major histocompatibility antigens on small cell carcinoma of the lung cell lines analyzed by cytofluorography: modulation by gamma-interferon. Cancer Res.

[34] Deng X, Ruvolo P, Carr B, May WS (2000). Survival function of ERK1/2 as IL-3-activated, staurosporine-resistant Bcl2 kinases. Proc Natl Acad Sci U S A.

[35] Chang KM, Chen SH, Kuo CJ, Chang CK, Guo RT, Yang JM, Liang PH (2012). Roles of amino acids in the Escherichia coli octaprenyl diphosphate synthase active site probed by structure-guided site-directed mutagenesis. Biochemistry.

[36] Mackenzie GG, Bartels LE, Xie G, Papayannis I, Alston N, Vrankova K, Ouyang N, Rigas B (2013). A novel Ras inhibitor (MDC-1016) reduces human pancreatic tumor growth in mice. Neoplasia.

[37] de Rooij J, Bos JL (1997). Minimal Ras-binding domain of Raf1 can be used as an activation-specific probe for Ras. Oncogene.

[38] Ostrem JM, Peters U, Sos ML, Wells JA, Shokat KM (2013). K-Ras(G12C) inhibitors allosterically control GTP affinity and effector interactions. Nature.

[39] Shima F, Yoshikawa Y, Ye M, Araki M, Matsumoto S, Liao J, Hu L, Sugimoto T, Ijiri Y, Takeda A (2013). In silico discovery of small-molecule Ras inhibitors that display antitumor activity by blocking the Ras-effector interaction. Proc Natl Acad Sci U S A.

[40] Jin L, Li D, Alesi GN, Fan J, Kang HB, Lu Z, Boggon TJ, Jin P, Yi H, Wright ER (2015). Glutamate dehydrogenase 1 signals through antioxidant glutathione peroxidase 1 to regulate redox homeostasis and tumor growth. Cancer Cell.

[41] You S, Li R, Park D, Xie M, Sica GL, Cao Y, Xiao ZQ, Deng X (2014). Disruption of STAT3 by niclosamide reverses radioresistance of human lung cancer. Mol Cancer Ther.

[42] Deng X, Gao F, Flagg T, Anderson J, May WS (2006). Bcl2's flexible loop domain regulates p53 binding and survival. Mol Cell Biol.

[43] Deng X, Xiao L, Lang W, Gao F, Ruvolo P, May WS (2001). Novel role for JNK as a stress-activated Bcl2 kinase. J Biol Chem.

[44] Kabeya Y, Mizushima N, Ueno T, Yamamoto A, Kirisako T, Noda T, Kominami E, Ohsumi Y, Yoshimori T (2000). LC3, a mammalian homologue of yeast Apg8p, is localized in autophagosome membranes after processing. EMBO J.

[45] Oltersdorf T, Elmore SW, Shoemaker AR, Armstrong RC, Augeri DJ, Belli BA, Bruncko M, Deckwerth TL, Dinges J, Hajduk PJ (2005). An inhibitor of Bcl-2 family proteins induces regression of solid tumours. Nature.

[46] Li R, Ding C, Zhang J, Xie M, Park D, Ding Y, Chen G, Zhang G, Gilbert-Ross M, Zhou W (2017). Modulation of Bax and mTOR for Cancer therapeutics. Cancer Res.

[47] Ji H, Ramsey MR, Hayes DN, Fan C, McNamara K, Kozlowski P, Torrice C, Wu MC, Shimamura T, Perera SA (2007). LKB1 modulates lung cancer differentiation and metastasis. Nature.

[48] Shackelford DB, Abt E, Gerken L, Vasquez DS, Seki A, Leblanc M, Wei L, Fishbein MC, Czernin J, Mischel PS, Shaw RJ (2013). LKB1 inactivation dictates therapeutic response of non-small cell lung cancer to the metabolism drug phenformin. Cancer Cell.

[49] Han B, Park D, Li R, Xie M, Owonikoko TK, Zhang G, Sica GL, Ding C, Zhou J, Magis AT (2015). Small-molecule Bcl2 BH4 antagonist for lung Cancer therapy. Cancer Cell.

[50] Park D, Magis AT, Li R, Owonikoko TK, Sica GL, Sun SY, Ramalingam SS, Khuri FR, Curran WJ, Deng X (2013). Novel small-molecule inhibitors of Bcl-XL to treat lung cancer. Cancer Res.

[51] Xin M, Li R, Xie M, Park D, Owonikoko TK, Sica GL, Corsino PE, Zhou J, Ding C, White MA (2014). Small-molecule Bax agonists for cancer therapy. Nat Commun.

[52] Yoshii Saori R., Mizushima Noboru (2017). Monitoring and Measuring Autophagy. International Journal of Molecular Sciences.

[53] Liang C, Feng P, Ku B, Dotan I, Canaani D, Oh BH, Jung JU (2006). Autophagic and tumour suppressor activity of a novel Beclin1-binding protein UVRAG. Nat Cell Biol.

[54] Jackson EL, Willis N, Mercer K, Bronson RT, Crowley D, Montoya R, Jacks T, Tuveson DA (2001). Analysis of lung tumor initiation and progression using conditional expression of oncogenic K-ras. Genes Dev.

[55] Tuveson DA, Shaw AT, Willis NA, Silver DP, Jackson EL, Chang S, Mercer KL, Grochow R, Hock H, Crowley D (2004). Endogenous oncogenic K-ras(G12D) stimulates proliferation and widespread neoplastic and developmental defects. Cancer Cell.

[56] Frese KK, Tuveson DA (2007). Maximizing mouse cancer models. Nat Rev Cancer.

[57] Hopkins AL, Groom CR (2002). The druggable genome. Nat Rev Drug Discov.

[58] Patricelli MP, Janes MR, Li LS, Hansen R, Peters U, Kessler LV, Chen Y, Kucharski JM, Feng J, Ely T (2016). Selective inhibition of oncogenic KRAS output with small molecules targeting the inactive state. Cancer Discov.

[59] Lito P, Solomon M, Li LS, Hansen R, Rosen N (2016). Allele-specific inhibitors inactivate mutant KRAS G12C by a trapping mechanism. Science.

[60] Smith G, Bounds R, Wolf H, Steele RJ, Carey FA, Wolf CR (2010). Activating K-Ras mutations outwith 'hotspot' codons in sporadic colorectal tumours - implications for personalised cancer medicine. Br J Cancer.

