# Correction to: Small Molecule KRAS Agonist for Mutant KRAS Cancer Therapy

**DOI:** 10.1186/s12943-020-01214-5

**Published:** 2020-05-20

**Authors:** Ke Xu, Dongkyoo Park, Andrew T. Magis, Jun Zhang, Wei Zhou, Gabriel L. Sica, Suresh S. Ramalingam, Walter J. Curran, Xingming Deng

**Affiliations:** 1 Division of Cancer Biology, Department of Radiation Oncology, Emory University School of Medicine and Winship Cancer Institute of Emory University, Atlanta, GA 30322 USA; 2 Institute for Systems Biology, Seattle, WA 98109 USA; 3 Division of Hematology, Oncology and Blood & Marrow Transplantation, Department of Internal Medicine, Holden Comprehensive Cancer Center, University of Iowa Carver College of Medicine, Iowa City, IA 52242 USA; 4 Department of Hematology and Medical Oncology, Emory University School of Medicine and Winship Cancer Institute of Emory University, Atlanta, GA 30322 USA; 5 Department of Pathology and Laboratory Medicine, Emory University School of Medicine and Winship Cancer Institute of Emory University, Atlanta, GA 30322 USA

**Correction to: Mol Cancer (2019) 18:85**

**https://doi.org/10.1186/s12943-019-1012-4**

Following the publication of the original article [1], the authors spotted error in Fig. 1C. The colony formation image of H1792 in control row was accidently placed the same image of H322. This is a mistake and has to be corrected. Now, the actual image of H1792 in control row is placed at H1792 spot in revised Fig. 1 (below). This correction does not change any conclusions made in the article. The authors apologize for this error and any confusion it may have caused.


