# Supplementary figures and images for: Small Molecule KRAS Agonist for Mutant KRAS Cancer Therapy
Source: Mol Cancer. 2019 Apr 10;18:85. doi: 10.1186/s12943-019-1012-4 (PMC6456974; doi:10.1186/s12943-019-1012-4)

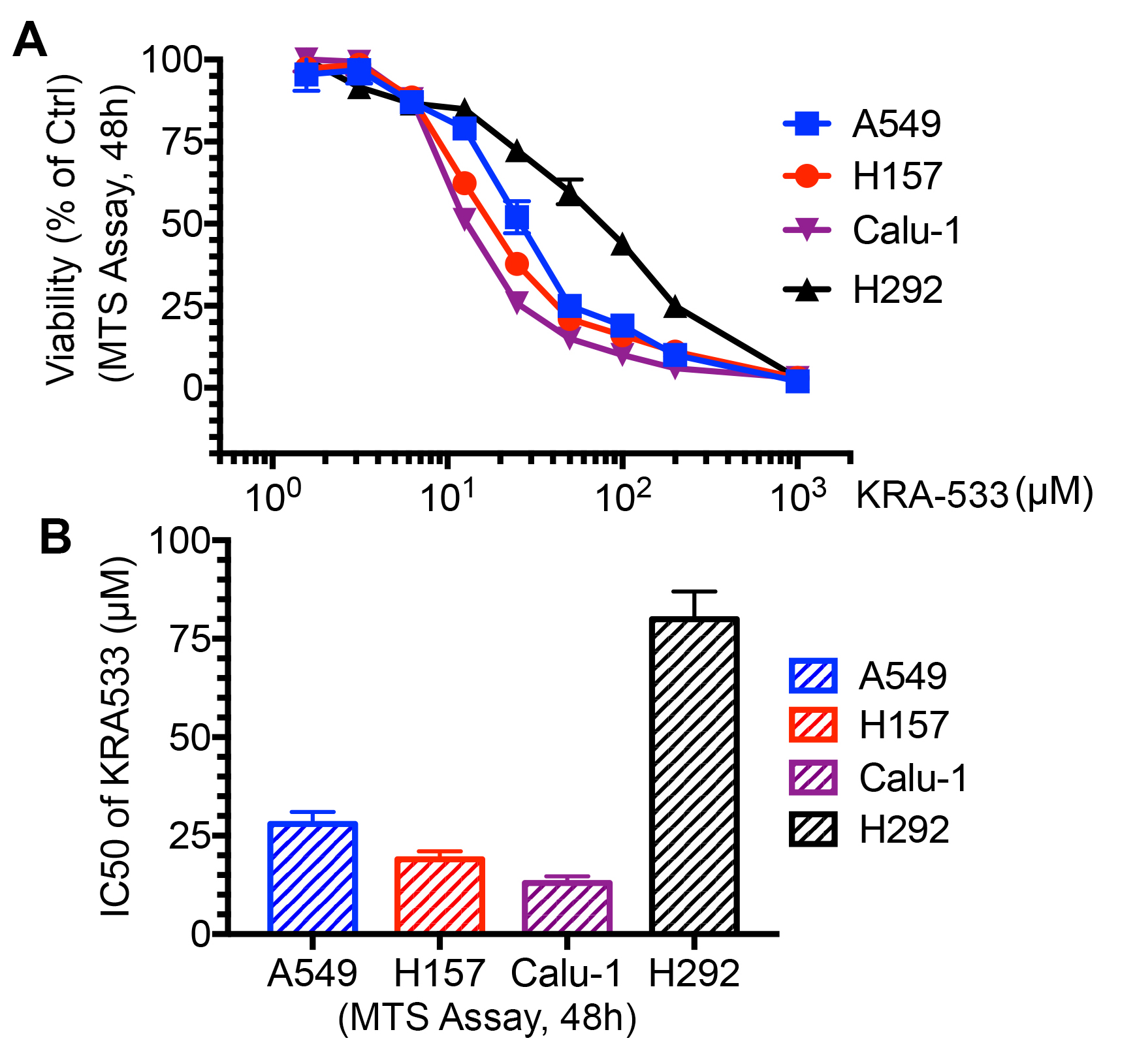

Supplement: Supplementary file 1 — Figure S1. KRA-533 inhibits proliferation of NSCLC cells. (A) A549, H157, Calu-1 and H292 cells were treated with increasing concentrations of KRA-533, followed by analysis of cell proliferation using MTS Cell Proliferation Colorimetric Assay Kit. (B) IC50 values of KRA533 based on cell proliferation data from (A). (JPG 700 kb) [file 12943_2019_1012_MOESM1_ESM.jpg]

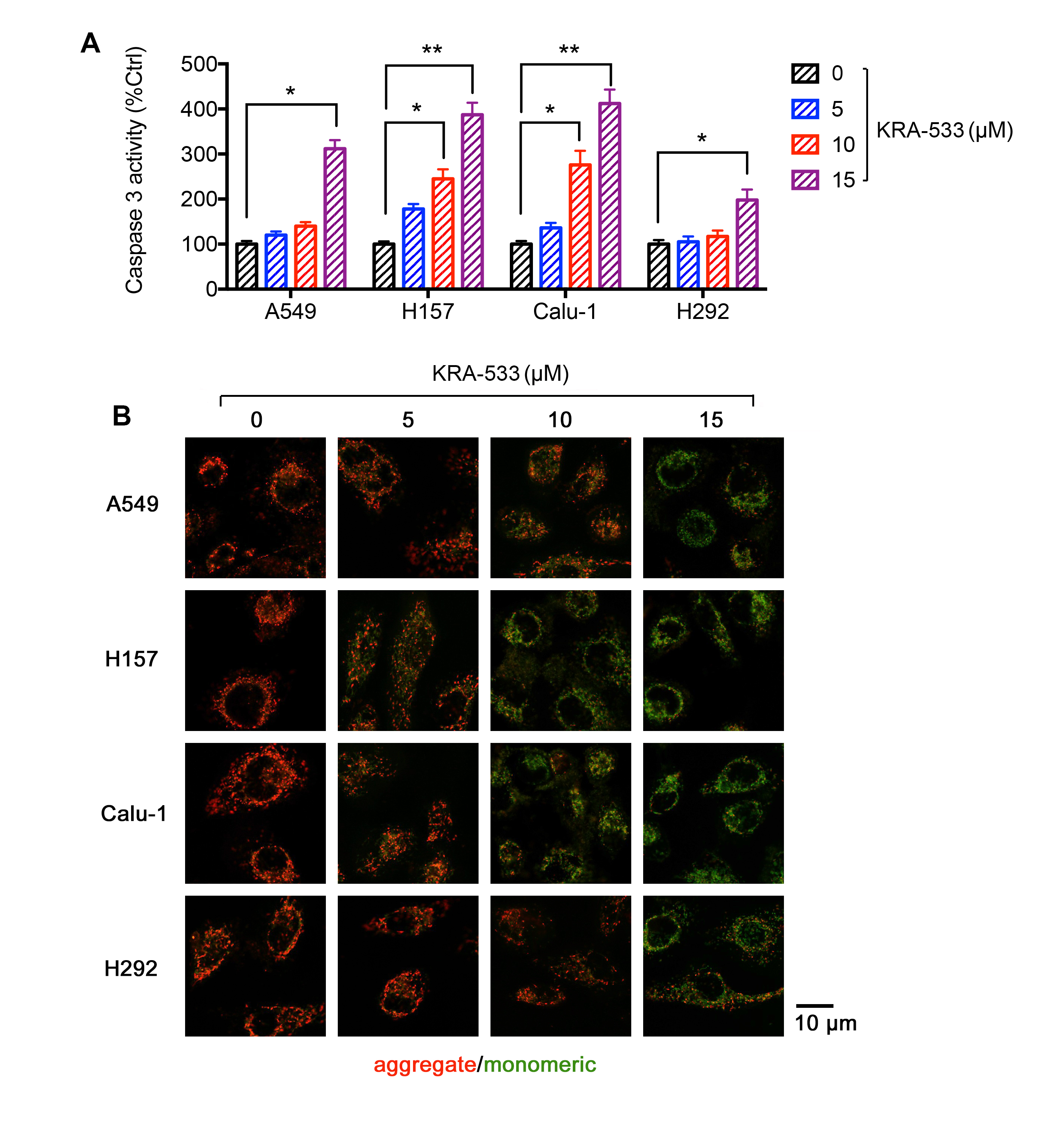

Supplement: Supplementary file 2 — Figure S2. KRA-533 induces caspase 3 activation and reduces mitochondrial membrane potential in NSCLC cells. (A) and (B) A549, H157, Calu-1 and H292 cells were treated with increasing concentrations of KRA-533, followed by analysis of caspase 3 activity using Caspase 3 Colorimetric Assay Kit (A) and measurement of mitochondrial membrane potential by JC-1 staining (B). Data represent mean ± SD, *P < 0.05, **P < 0.01, by 2-tailed t test. (JPG 2498 kb) [file 12943_2019_1012_MOESM2_ESM.jpg]

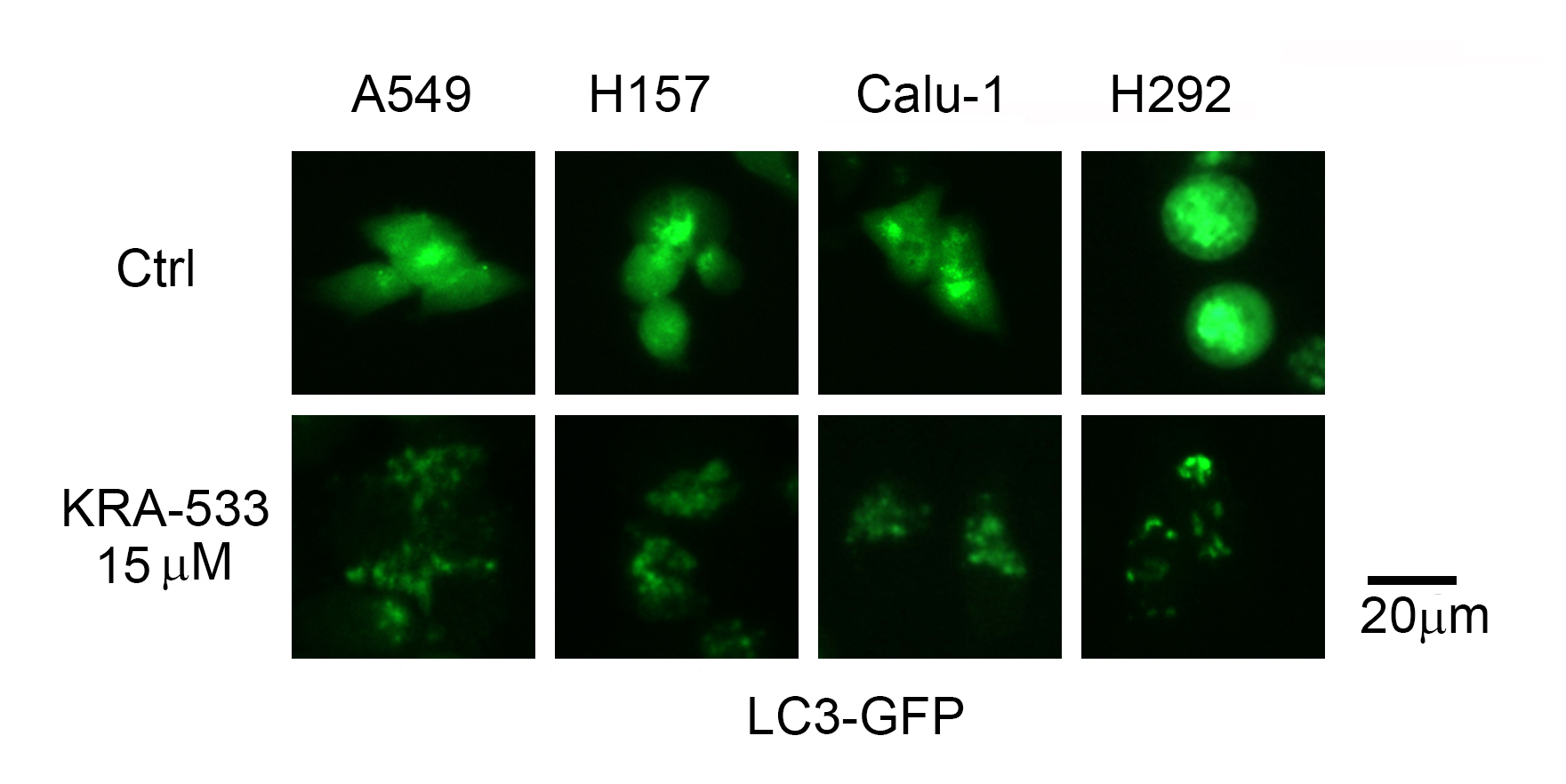

Supplement: Supplementary file 3 — Figure S3. KRA-533 induces autophagy formation in NSCLC cells. A549, H157, Calu-1 and H292 cells were transfected with GFP-LC3. After 24 h, cells were treated with KRA-533 for 48 h. Autophagic vacuoles in the representative cells from various treatments were shown. Scale bar represents 20 μm. (JPG 354 kb) [file 12943_2019_1012_MOESM3_ESM.jpg]

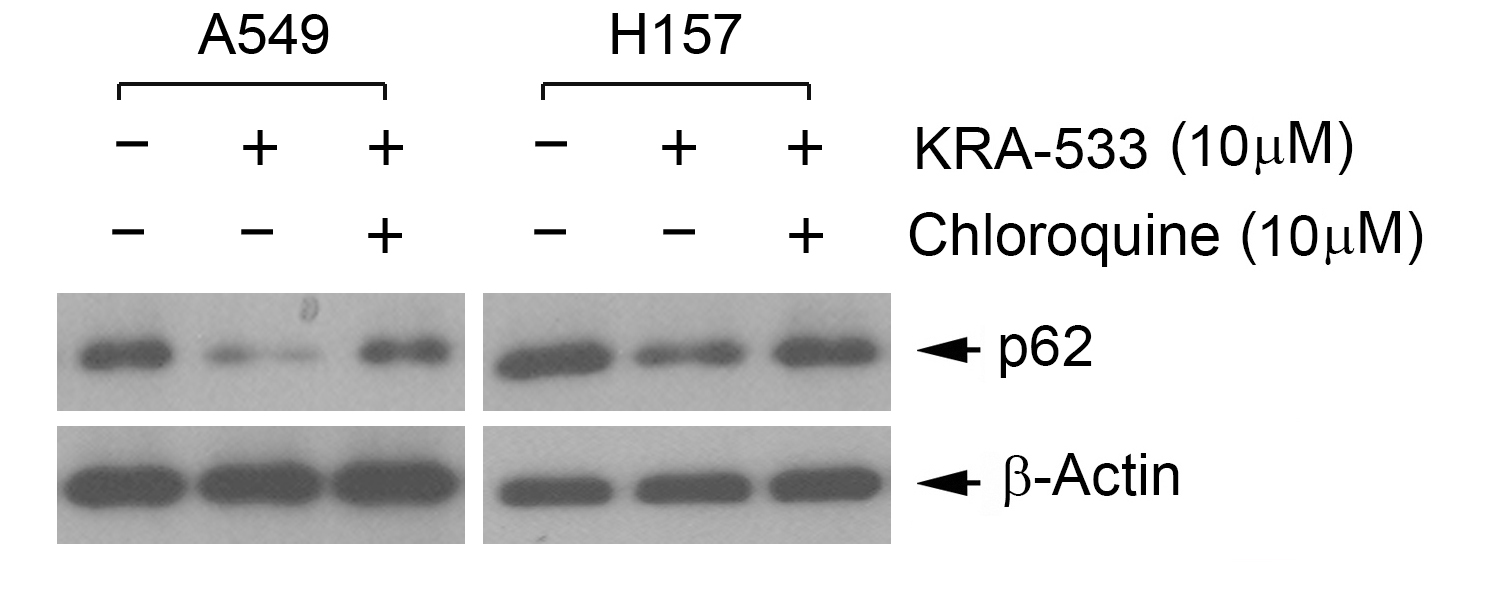

Supplement: Supplementary file 4 — Figure S4. The autophagy inhibitor chloroquine blocks KRA-533-induced autophagy in NSCLC cells. A549 and H157 cells were treated with KRA-533 (10 μM) in the absence or presence of autophagy inhibitor chloroquine (10 μM) for 48 h, followed by Western blot analysis of the autophagy marker p62. (JPG 187 kb) [file 12943_2019_1012_MOESM4_ESM.jpg]

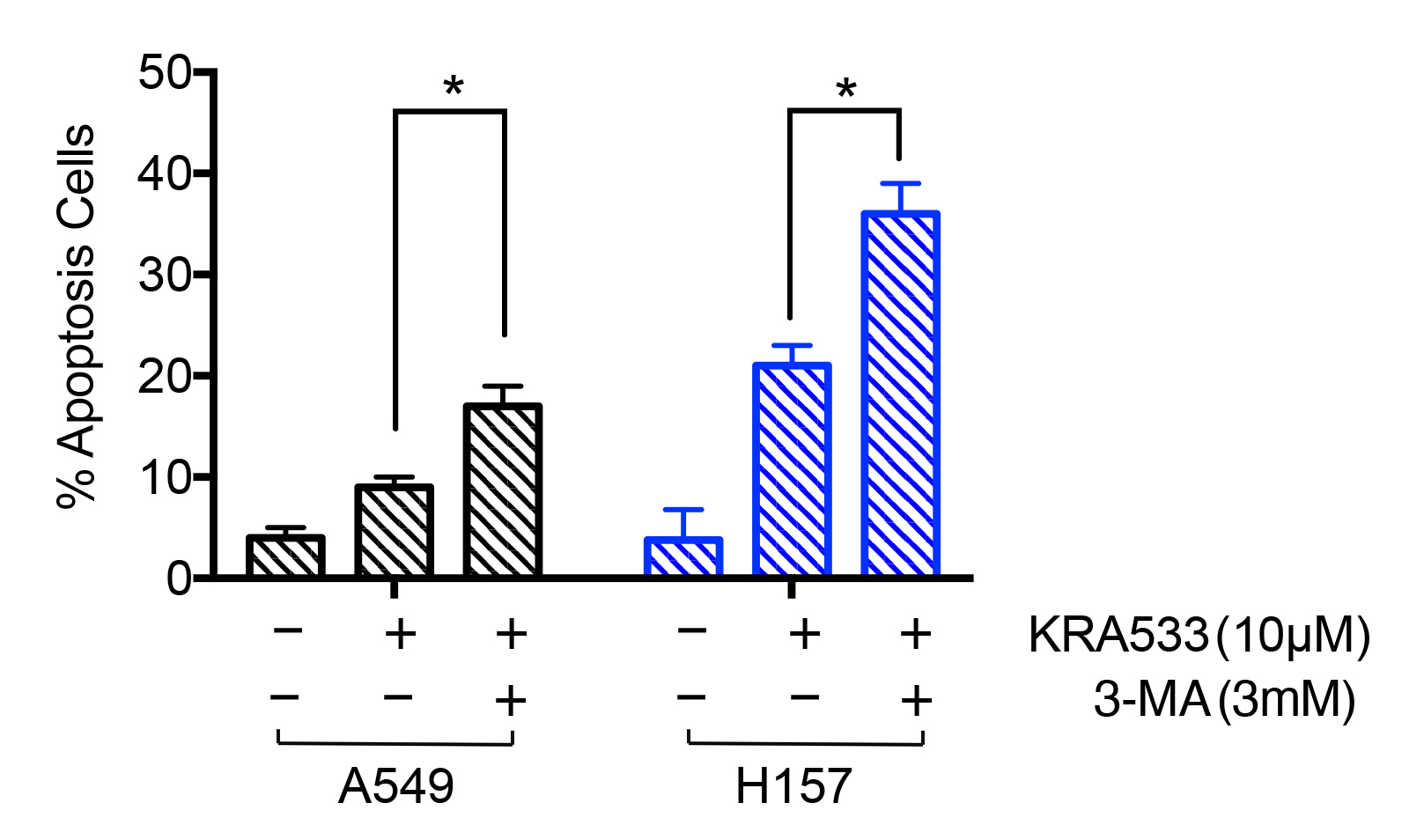

Supplement: Supplementary file 5 — Figure S5. The autophagy inhibitor 3-methyladenine (3-MA) enhanced KRA-533-induced apoptosis of NSCLC cells. A549 and H159 cells were treated with KRA-533 (10 μM) in the absence or presence of 3-MA for 48 h, followed by FACS analysis of Annexin V/PI staining for apoptosis. Data represent mean ± SD, *P < 0.05, by 2-tailed t test. (JPG 362 kb) [file 12943_2019_1012_MOESM5_ESM.jpg]

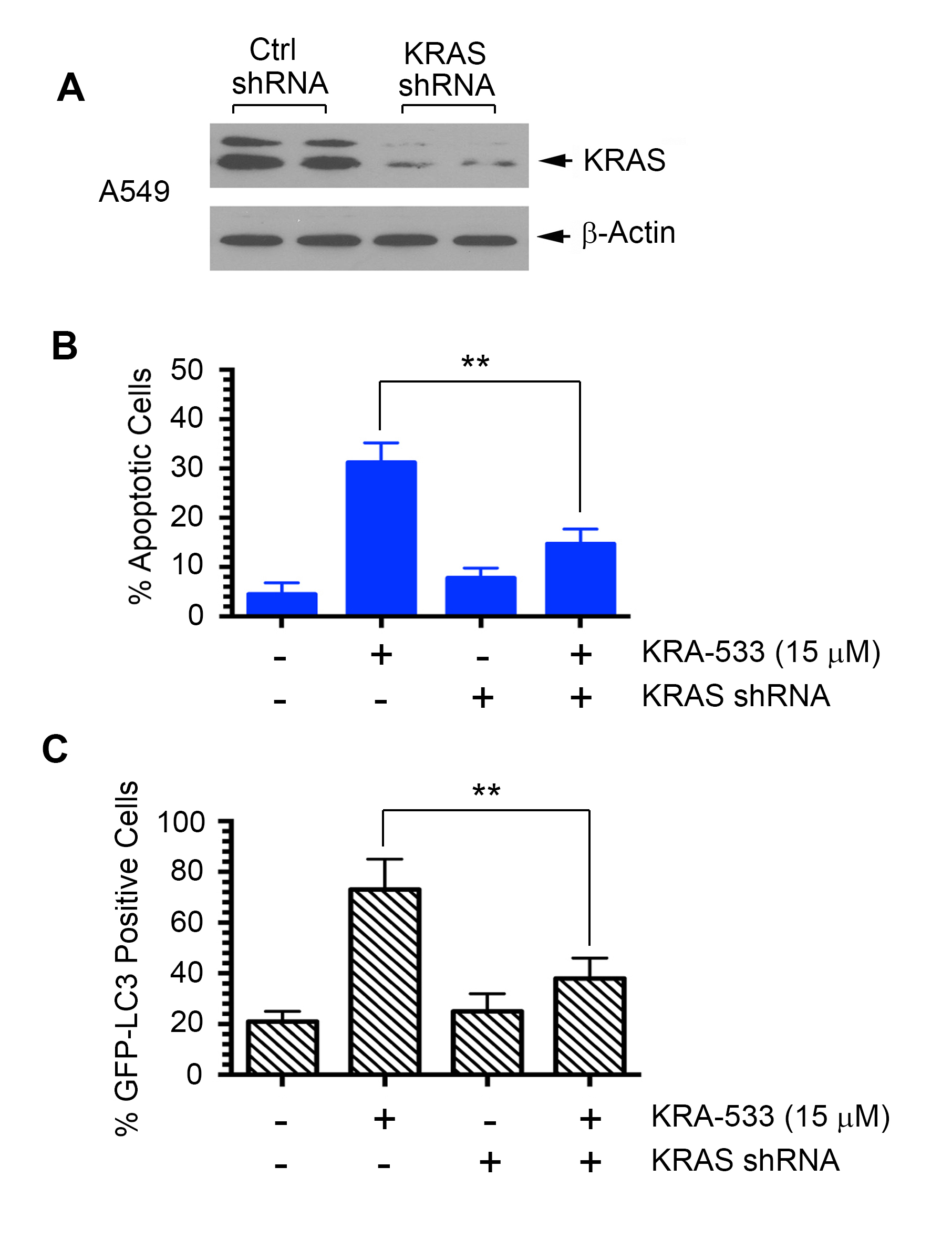

Supplement: Supplementary file 6 — Figure S6. Silencing of mutant KRAS reduced sensitivity of cells to KRA-533. (A) KRAS shRNA plasmids were transfected into A549 cells that contain KRAS mutation, followed by Western blot using KRAS antibody. (B) A549 cells and mutant KRAS silenced A549 cells were treated with KRA-533 (15 μM) for 48 h. Apoptotic cells were detected by Annexin V /PI binding and analyzed by FACS. Data represent mean ± SD, **P < 0.01, by 2-tailed t test. (C) GFP-LC3 constructs and KRAS shRNA plasmids were co-transfected into A549 cells, followed by treatment with KRA-533 for 48 h. Autophagic cells (GFP-LC3 positive cells) were visualized by Axioplan Zeiss microscope and quantified. Data represent mean ± SD, **P < 0.01, by 2-tailed t test. (JPG 653 kb) [file 12943_2019_1012_MOESM6_ESM.jpg]

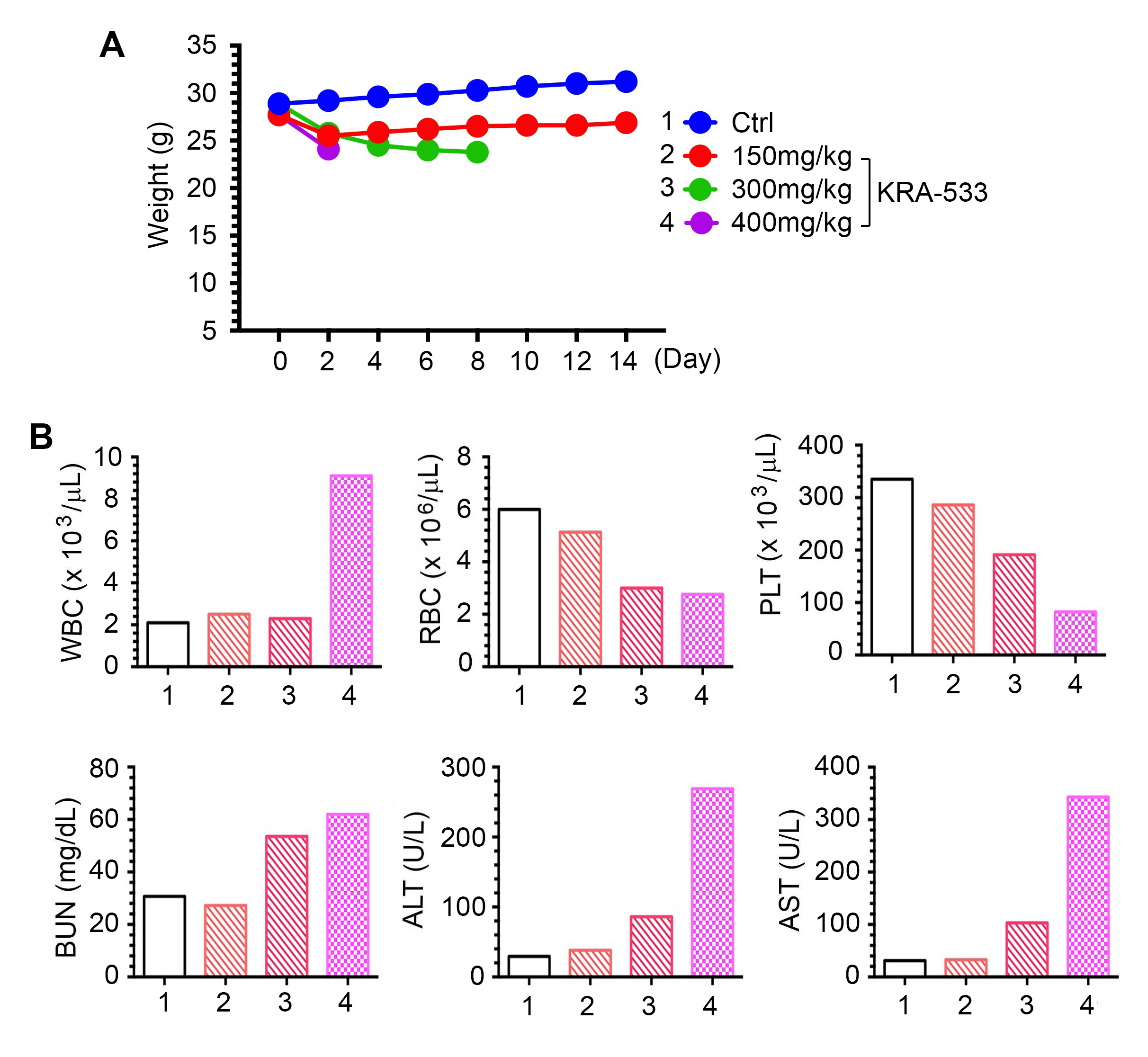

Supplement: Supplementary file 7 — Figure S7. Determination of single dose maximum tolerated dose (MTD). (A) Nu/Nu nude mice were treated with single dose (i.e. 0, 150 mg/kg, 300 mg/kg or 400 mg/kg) KRA-533 via i.p. (n = 6 mice per group). After treatment, the body weight of mice was measured once every other day for 2 weeks. (B) Blood analysis of mice after treatment with single dose(s) of KRA-533. (C) H&E histology of various organs from mice after treatment with single dose(s) of KRA-533. (JPG 1446 kb) [file 12943_2019_1012_MOESM7_ESM.jpg]

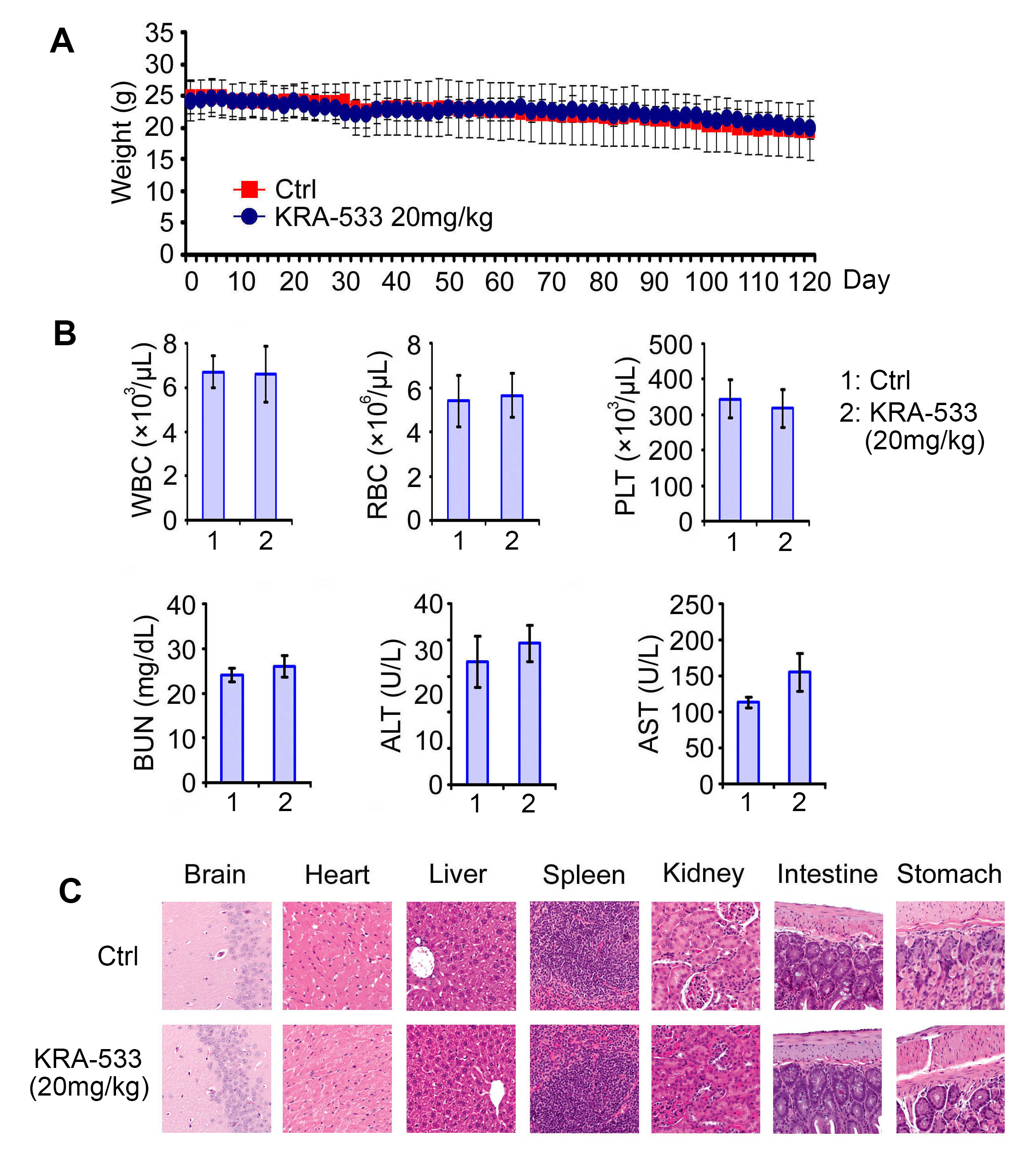

Supplement: Supplementary file 8 — Figure S8. Toxicity of KRA-533 in genetically engineered LSL-KRAS G12D mice. (A), (B) and (C), Body weight, blood analysis and H&E histology of various organs from mice bearing A549 xenografts after treatment with KRA-533 (20 mg/kg/d) for 4 months. (JPG 2133 kb) [file 12943_2019_1012_MOESM8_ESM.jpg]

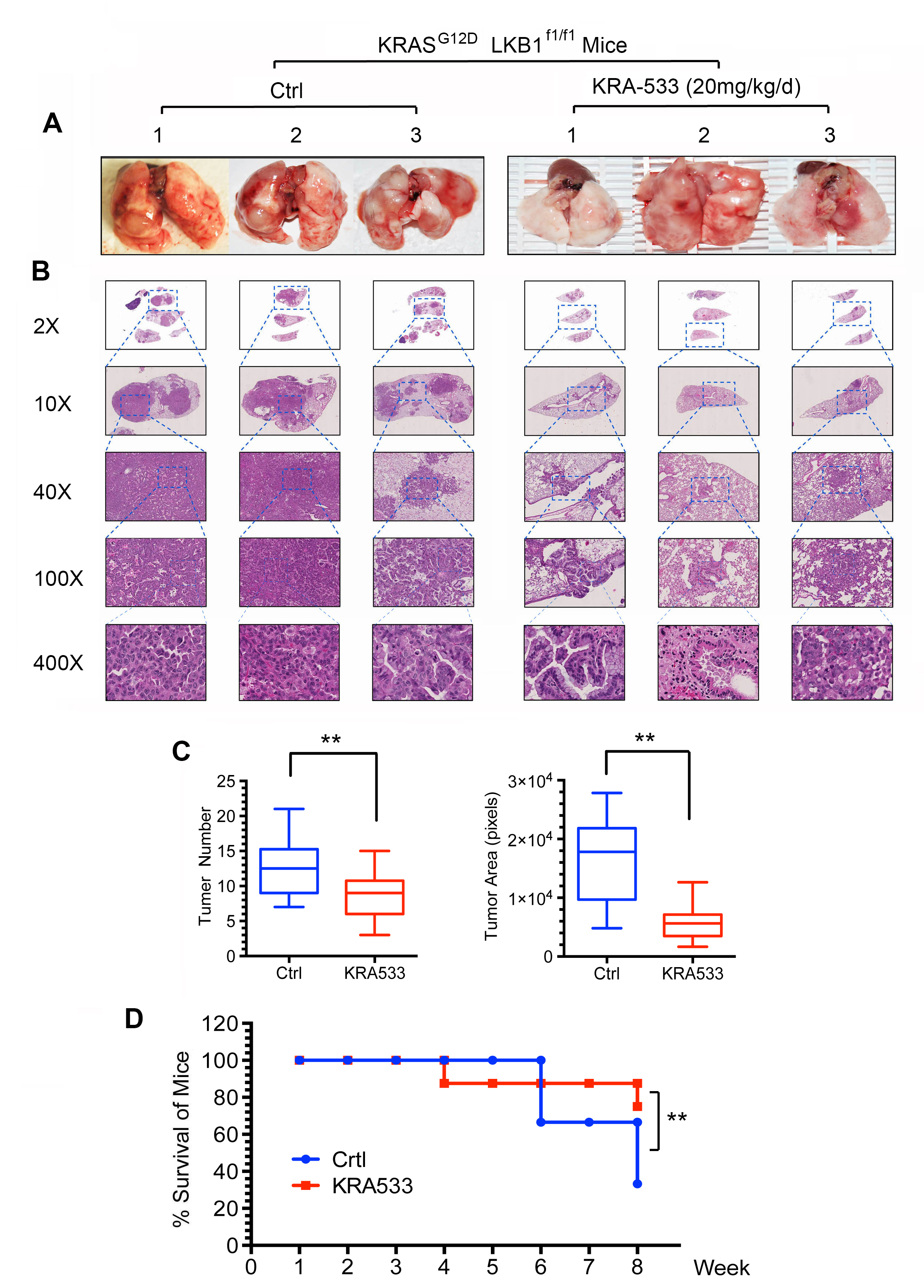

Supplement: Supplementary file 9 — Figure S9. Potency of KRA-533 in genetically engineered LSL-KRASG12D LKB1fl/fl (KL) mice. (A) and (B) After administration of adenovirus Cre recombinase in KL mice for 6 weeks, mice were treated with KRA-533 (20 mg/kg/d) for 8 weeks (n = 6 each group). Three representative brightfield images of mouse lungs and H&E images from control or KRA-533 treatment group are shown. (C) Tumor numbers were counted under the microscope and tumor area was quantified using Openlab modular imaging software. Data represent the mean ± SD, n = 6 per group. **P < 0.01, by 2-tailed t test. (D) Survival of mice was calculated up to 8 weeks before euthanization in the control group versus the KRA-533 treatment group. Data represent mean ± SD, n = 6 per group. **P < 0.01, by 2-tailed t test. (JPG 3111 kb) [file 12943_2019_1012_MOESM9_ESM.jpg]
